# Reversible Kinetic Trapping of FUS Biomolecular Condensates

**DOI:** 10.1002/advs.202104247

**Published:** 2021-12-04

**Authors:** Sayantan Chatterjee, Yelena Kan, Mateusz Brzezinski, Kaloian Koynov, Roshan Mammen Regy, Anastasia C. Murthy, Kathleen A. Burke, Jasper J. Michels, Jeetain Mittal, Nicolas L. Fawzi, Sapun H. Parekh

**Affiliations:** ^1^ Department of Biomedical Engineering University of Texas at Austin 107 W. Dean Keeton Rd. Austin TX 78712 USA; ^2^ Max Planck Institute for Polymer Research Ackermannweg 10 Mainz 55128 Germany; ^3^ LUT School of Engineering Science LUT University Yliopistonkatu 34 Lappeenranta 53850 Finland; ^4^ Artie McFerrin Department of Chemical Engineering Texas A&M University 200 Jack E. Brown Engineering Building College Station TX 77843 USA; ^5^ Department of Molecular Biology, Cell Biology, and Biochemistry Brown University 70 Ship Street Providence RI 02912 USA

**Keywords:** biointerphases, biomolecular condensates, fused in sarcoma, molecular spectroscopy, phase separation

## Abstract

Formation of membrane‐less organelles by self‐assembly of disordered proteins can be triggered by external stimuli such as pH, salt, or temperature. These organelles, called biomolecular condensates, have traditionally been classified as liquids, gels, or solids with limited subclasses. Here, the authors show that a thermal trigger can lead to formation of at least two distinct liquid condensed phases of the fused in sarcoma low complexity (FUS LC) domain. Forming FUS LC condensates directly at low temperature leads to formation of metastable, kinetically trapped condensates that show arrested coalescence, escape from which to untrapped condensates can be achieved via thermal annealing. Using experimental and computational approaches, the authors find that molecular structure of interfacial FUS LC in kinetically trapped condensates is distinct (more *β*‐sheet like) compared to untrapped FUS LC condensates. Moreover, molecular motion within kinetically trapped condensates is substantially slower compared to that in untrapped condensates thereby demonstrating two unique liquid FUS condensates. Controlling condensate thermodynamic state, stability, and structure with a simple thermal switch may contribute to pathological protein aggregate stability and provides a facile method to trigger condensate mixing for biotechnology applications.

## Graphical abstract

Cold formation of intrinsically disordered fused in sarcoma condensates creates metastable, kinetically trapped liquid condensates (A) that can be converted into equilibrium liquid condensates (C) via thermal annealing (B). Multiple liquid states allow for on‐demand, stimuli‐responsive mixing of condensate contents to control composition dynamically.

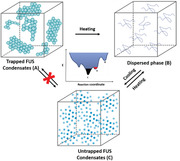

## 1 Introduction

Eukaryotic cellular organization, once thought to be exclusively dominated by membrane‐enveloped organelles, has been reimagined over the last decade following numerous demonstrations of liquid‐like, membraneless compartments in cells. Perhaps the most well‐studied protein shown to undergo condensation is FUS, an RNA‐binding protein that assembles into ribonucleoprotein particles such as stress granules formed, at least in part, by liquid–liquid phase separation (LLPS).^[^ ^1^ ^]^ Phase separation of FUS has been shown to result in formation of membraneless organelles, known as biomolecular condensates (BCs), that are believed to have multiple physiological roles from biomaterial sequestration to regulating transcription and translation.^[^ ^2^ ^]^ BC formation can be recapitulated in vitro from purified FUS and results in two distinct phases: a protein‐rich (“droplet”) phase suspended in a protein‐depleted phase. Recent work has shown that additional macromolecules such as RNA and other transcription factors are also enriched in the protein‐dense phase.^[^ ^3^ ^]^ A conserved feature in FUS, and many other BC forming proteins, is the presence of intrinsically disordered regions (IDRs) in the protein sequence for which no defined secondary structure has been observed.^[^ ^4^ ^]^ The N‐terminal portion of FUS contains a low‐complexity domain (LCD), FUS LC, that is an IDR enriched with glycine and uncharged polar amino acids, which undergoes condensation.^[^ ^5^ ^]^

FUS predominantly localizes to the nucleus where it is involved in gene transcription and regulation, DNA repair, RNA shearing, RNA transport, translation, and maintenance of genomic stability.^[^ ^6^ ^]^ Mutations in the FUS LC are known to correlate with formation of irreversible pathological aggregates, which have been found in various neurodegenerative diseases such as amyotrophic lateral sclerosis and frontal temporal dementia.^[^ ^7^ ^]^ Indeed, the uncharged, polar LCD in FUS promotes the formation of membraneless liquid‐like condensates, which can convert to static structures with time.^[^ ^1b^ ^]^ Therefore, understanding how different material states of FUS—liquid‐like, gel‐like, or pathological aggregates—are stabilized is of paramount interest to differentiate between normal granule formation and pathogenic aggregation. In particular, elucidating how the interfacial properties of FUS condensates contribute to coalescence or agglomeration behavior is of high interest but has remained unclear.

Thus far, truncations of FUS containing at least the LCD have been shown to form three types of thermodynamically reversible states in vitro: soluble protein solutions, liquid droplets, and hydrogels.^[^ ^1^, ^8^, ^10^ ^b]^ These states have been extensively characterized in bulk. Solution NMR studies and vibrational spectroscopy have shown that FUS LC (amino acids 1–163) is disordered when dissolved in solution and remains disordered in liquid‐like condensates.^[^ ^9^ ^]^ FUS LC (amino acids 1–214) hydrogels, which are both thermodynamically and kinetically distinct from liquid‐like condensates, form over days while liquid‐like condensates form in seconds. FUS LC in hydrogels shows a distinct *β*‐sheet structure in contrast to the disordered protein structure in liquid droplets.^[^ ^5^, ^10^ ^]^ Weak, noncovalent interactions such as electrostatic attraction, *π*–*π*, cation–*π*, H‐bonding, and hydrophobic interactions are thought to promote both types of condensate formation.^[^ ^9^, ^11^ ^]^ Therefore, external stimuli (pH, temperature, protein concentration, salt concentration, etc.) may be relatively impactful for modifying the phase separation of FUS LC.^[^ ^5^, ^11^, ^12^ ^]^

We note while FUS LC and other IDRs have garnered significant attention for their phase separation properties via weak interactions, a disordered region is certainly not a prerequisite for a protein to exhibit LLPS. In fact, LLPS has been shown to occur for folded proteins well before the current interest in phase transitions of disordered proteins and IDRs.^[^ ^13^ ^]^ Mechanistically however, LLPS of a disordered protein may be different from that of a folded protein. A phase separating IDR typically contains multiple amino acid sequences that act as mutually attractive sites, which are well accessible owing to the flexibility associated with the lack of a secondary structure. In this situation, LLPS is induced by multivalency^[^ ^14^ ^]^ and the IDR remains disordered,^[^ ^5b^ ^]^ irrespective of whether it resides in the concentrated or the dilute phase. In contrast, LLPS of folded proteins is often accompanied by a conformational change, giving rise to an additional entropic gain by expelling water molecules to the bulk solution.^[^ ^15^ ^]^

With many of the fundamental interactions for IDR protein condensation enumerated, significant attention has recently been focused on how to tune or control BCs. In this spirit, Schuster et al. showed that it was ossible to build designer condensates using fusion proteins of an IDR to other “cargo” proteins.^[^ ^16^ ^]^ Building on this finding, Reinkemeier et al. showed that it was possible to assemble entire functioning organelles—in their case an orthogonal ribosome—based on a similar concept of fusing FUS LC or other IDRs to proteins required for protein translation.^[^ ^17^ ^]^ While controlling the contents of BCs is undoubtedly critical for both learning more about BCs/compartmentalization in cells and applications in biotechnology, identifying stimuli‐responsive BC properties is equally important. What are the methods to stabilize BCs against mixing? Can stabilized condensates be triggered to mix? These questions have received comparatively little study.

Since temperature can be easily and rapidly modulated, there is a large interest in thermoresponsive LLPS applications.^[^ ^18^ ^]^ Murakami et al. showed that FUS LC (residues 2–214) could be reversibly, thermally converted from a dissolved phase to a gel phase multiple times before irreversible gelling occurred.^[^ ^10b^ ^]^ Similarly, other peptides such as proline–arginine dipeptide repeats or elastin‐ and resilin‐like peptides have also been shown to exhibit multiple thermally labile LLPS transitions and morphologies.^[^ ^19^ ^]^ Despite these demonstrations, the molecular organization in condensates inhabiting states with different morphologies is unknown and challenges remain in controllably triggering condensate mixing (in addition to disassembly) with thermal stimuli.

In this study, we report that formation of FUS LC (residues 1–163) condensates in the cold creates discrete, kinetically trapped BCs that show arrested coalesce, which can be thermally converted into untrapped condensates that readily coalesce. The contents of individually prepared kinetically trapped BCs, which do not mix, could be triggered to mix as untrapped condensates by thermal annealing. Using fluorescence and molecular imaging and spectroscopy together with simulations, we quantified the structural diversity and physical properties of FUS LC kinetically trapped and kinetically untrapped BCs.

## 2 Results and Discussion

### 2.1 Kinetic Trapping of FUS LC in Biomolecular Condensates

**Figure 1:**
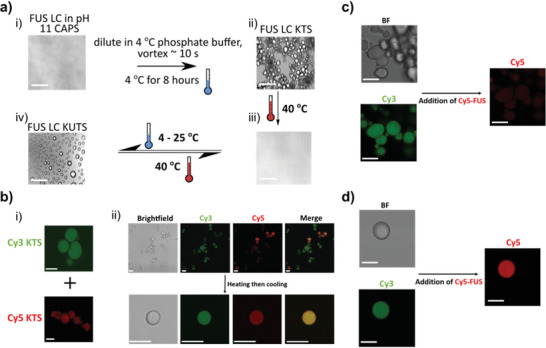
Formation of trapped (KTS) and untrapped (KUTS) FUS LC condensates. a) Formation of kinetically trapped and untrapped FUS LC condensates by cold incubation. Brightfield microscopy of 1 mm FUS LC in CAPS at pH 11 (i). Dilution of FUS LC to 200 µm with 4 °C phosphate buffer at pH 7.4, brief vortexing, and refrigeration resulted in KTS condensates (ii); heating to 40 °C led to redissolved FUS LC (iii); and cooling to 4–25 °C led to KUTS condensates (iv). Switching between formation of KUTS condensates and fully dissolved FUS LC was thermally reversible. Scale bars are 5 µm. b) Brightfield and fluorescence images of separately prepared Cy5 and Cy3 KTS BCs containing 0.01% labeled protein before mixing into a single sample (i). After mixing, the sample was imaged before (top row) and after (bottom row) thermal annealing (ii). Scale bars are 10 µm. c) KTS condensates (Cy3 labeled) almost completely excluded soluble Cy5‐FUS LC added after formation. Scale bars are 10 µm. d) KUTS condensates (Cy3 labeled) fully incorporate soluble Cy5‐FUS LC added after formation. Scale bars are 10 µm. Imaging and display parameters for (c) and (d) are identical. All microscopy was performed at room temperature.

The QGSY‐rich region of the FUS (residues 1–163), here after referred to as FUS LC, shows an upper critical solution temperature‐mediated phase transition into droplet‐like BCs via LLPS at room temperature, in physiological buffers, when the concentration is above ≈150 µm.^[^ ^5b^ ^]^ By preparing a 200 µm FUS LC solution in 4 °C pH 7.4 phosphate buffer,^[^ ^5b^ ^]^ vortexing for 5–10 s, and storing the solution at 4 °C overnight, the next morning we observed nonfusing condensates that we call kinetically trapped states (KTS) of FUS LC (**Figure** **1**). In contrast to well‐studied FUS LC liquid droplets that fuse upon contact, KTS FUS LC condensates formed assemblies of condensates juxtaposed to one another that remained distinct during room temperature observation (Figure 1a[ii]). By raising the temperature to 40 °C, we found KTS FUS LC condensates completely dissolved, and the solution became transparent as a single phase (Figure 1a[iii]). Surprisingly, by allowing the solution to cool to room temperature (≈23 °C) by natural convention, familiar liquid‐like droplets—that fused—appeared. We call these droplet condensates kinetically untrapped states (KUTS) of FUS LC (Figure 1a[iv] and Figure S1, Supporting Information). Interestingly, we found that originally separate KTS condensates, made with (0.01 mol%) Cy3‐labeled FUS LC (Cy3‐FUS LC) or Cy5‐labeled FUS LC (Cy5‐FUS LC), appeared mixed in KUTS condensates after this annealing process (Figure 1b). Thermal annealing thus enabled escape from KTS FUS LC condensates into KUTS FUS LC condensates.

We repeated the heating (to 40 °C) and cooling (to 23 °C) cycle up to seven times and repeatedly observed solution homogenization and KUTS formation, suggesting an equilibrium transition between dissolved FUS LC and KUTS condensates. In contrast, it was not possible to reform KTS FUS LC condensates once the solution was annealed, even by prolonged cooling (for days) at 4 °C, suggesting that KTS condensates are out of equilibrium condensates that are metastable. We note that cold formation of KTS of FUS LC condensates was robust, occurring 100% of the time after verifying “canonical” LLPS of FUS LC into liquid droplets as done in Burke et al.^[^ ^5b^ ^]^ Annealing of KTS into a homogeneous solution, and subsequent KUTS formation, was always possible with KTS condensates. Repeatability was also verified with proteins produced by multiple labs on different sides of the world.

We further explored the stability of KTS condensates against in situ dilution and KTS formation as a function of protein concentration, cooling temperature, and cooling time. Formation of agglomerated KTS FUS LC condensates was stable against in situ dilution down to 3 µm, and these condensates could be formed with a starting protein concentration as low as 25 µm (see Figure S1c, Supporting Information). In contrast, FUS LC KUTS condensates were not resistant to dilution nor did they stably form at concentrations below 150 µm, similar to canonical FUS LC droplets formed under similar solution conditions at room temperature.^[^ ^5b^ ^]^ This suggests that protein–protein interactions were comparatively stronger in KTS since they were more resilient against dilution. Studying KTS formation as a function of temperature and time, we found that KTS condensates were observed at temperatures as high as 15 °C for overnight incubations and showed arrested coalescence within ≈15 min of incubation at 4 °C (Figure S2c and Movie S1, Supporting Information). Comparing the size of KTS condensates at different temperatures for overnight formation, we found that increasing formation temperature led to smaller condensates. Thus, KTS condensates formed at 4 °C were the largest with a characteristic linear size of (≈44 µm) while those formed at 15 °C were the smallest (≈9 µm) (Figure S2d, Supporting Information).

With KTS condensates exhibiting arrested fusion and thus maintaining barriers between juxtaposed condensates, we wondered if these condensates exhibited a relatively hydrophobic or hydrophilic interface compared to that of KUTS condensates. We used multiple solvatochromic dyes, including Nile Red, to probe for the hydrophobicity/hydrophilicity of KTS compared to KUTS. Nile Red emission generally shows longer (red‐shifted) emission wavelengths in hydrophilic environments compared to hydrophobic environments.^[^ ^20^ ^]^ For KTS samples, Nile Red concentrated in the interfacial regions of the condensates. The emission of Nile Red from the KTS interface was more intense compared to the bulk and showed a consistently blue shifted peak emission wavelength (626 nm) compared to that in the center of the KTS condensates (emission peak at 635 nm). In contrast, the Nile Red‐infused KUTS condensates showed nearly uniform emission intensity and emission spectra across the entire condensate, with the peak emission wavelength being slightly longer than observed for the KTS bulk (639 nm, Figure S3a,b, Supporting Information). The shorter peak emission wavelength of Nile Red on the KTS condensate interface compared to the bulk indicates that the interface of KTS condensates was more hydrophobic than the center. As the fluorescence distribution and emission wavelength was approximately constant in KUTS FUS LC condensates, this indicates the KUTS condensate had similar hydrophobicity at the interface and in the bulk (Figure S3c,d, Supporting Information). We crosschecked these results using another solvatochromic dye called ANS.^[^ ^21^ ^]^ The results from ANS also showed that interface of KTS condensates was more hydrophobic than the bulk and that the interface and bulk of the KUTS condensates showed similar hydrophobicity (Figure S4, Supporting Information).

### 2.2 Interfacial Permeability, Protein Secondary Structure, and Protein–Protein Interaction Are Distinct in Kinetically Trapped FUS LC Condensates

KTS and KUTS FUS LC condensates, differing only by a low formation temperature, were clearly distinct and have different interfacial hydrophobicities. We further investigated the interfacial properties of the two condensates by forming KTS and KUTS condensates labeled with 0.01 mol% Cy3‐FUS LC and tested for exchange/entry of soluble Cy5‐FUS LC. KTS condensates showed almost complete exclusion of Cy5‐FUS LC over a 30‐min period whereas KUTS showed complete mixing of Cy3‐FUS LC and Cy5‐FUS LC (Figure 1c,d and Figure S5, Supporting Information). This result shows that the KTS and KUTS condensate interfaces have different permeability, in addition to hydrophobicity, suggesting that the molecular structure of proteins at the interface of condensates could be distinct from one another.

Next, we used a combination of circular dichroism (CD) and spatially resolved broadband coherent anti‐Stokes Raman scattering (BCARS) to probe the protein secondary structures and molecular interactions of the two condensate assemblies. CD spectra of FUS LC KTS condensate showed a large negative ellipticity feature at 215 nm.^[^ ^22^ ^]^ KUTS condensates showed a decrease of the negative feature at 215 nm and a slight red shift of the negative peak to ≈217 nm compared to KTS condensates (Figure S6, Supporting Information). Via spectral decomposition, we found that CD measurements showed a slight shift toward more *β*‐sheet conformation for KTS compared to KUTS condensates.^[^ ^23^ ^]^ Unfortunately, the lack of spatial resolution in CD measurements made it difficult to attribute the changes in protein structure to proteins in the KTS/KUTS condensates versus proteins in the buffer or to determine protein structure at condensate interfaces. Therefore, we used hyperspectral BCARS microscopy of KTS and KUTS, which we have previously used to study canonical FUS LC droplets,^[^ ^9^ ^]^ to spatially resolve the vibrational (Raman) fingerprints of FUS LC KTS and KUTS condensates.

**Figure 2:**
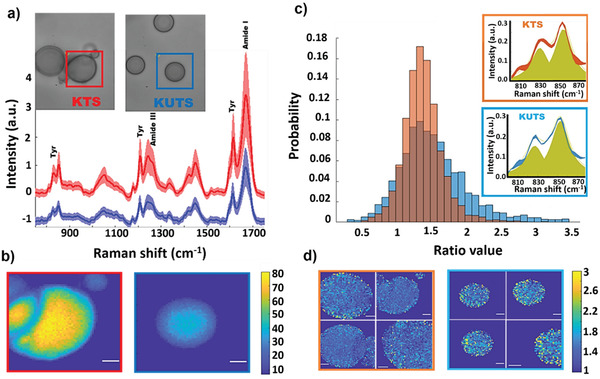
In situ BCARS imaging of protein–protein interactions in KTS/KUTS condensates. a) Fingerprint region spectra of KTS (red) and KUTS (blue). Shaded areas indicate the standard deviations (S.D.) of all the pixels within the measured sample area and solid line is the average. Insets show the brightfield images of measured areas. b) BCARS images of KUTS and KTS condensates boxed in (a) with same color coding. Pixel contrast is calculated from the Amide I integrated intensity and the pixel size is 0.3 × 0.3 µm^2^; color bar shows the Amide I intensity value in arbitrary units. Scale bar is 3 µm. c) Histogram of the tyrosine ratio (*I*_850_/*I*_830_) for KUTS and KTS. Data are from individual pixels pooled across four samples of each type. Histograms are area‐normalized such that the integrated probability is 1. Insets show tyrosine doublet average spectra with fitted peaks (yellow) and the S.D. between samples is shown by the width of the red or blue lines. d) BCARS images of four KTS (framed in orange) and four KUTS samples (framed in blue). Pixel contrast is calculated by tyrosine doublet integrated intensity ratio, and the color bar shows the ratio value from 1 to 3. Scale bar is 3 µm. All microscopy was performed at room temperature.

**Figure** **2**a shows the Raman vibrational spectrum from the boxed regions of KTS and KUTS condensates. The fingerprint region (800–1800 cm^−1^) consists of several pronounced bands, including tyrosine peaks at 830, 850, 1174, 1210, and 1616 cm^−1^, Amide I and Amide III bands from 1220 to 1250 cm^−1^ and 1630 to 1690 cm^−1^, and a CH_2_ peak at 1445 cm^−1^ (Figure 2a). The integrated intensity of the Amide I band, which is an indication of the concentration of protein in the sample, was approximately twofold higher in KTS compared to KUTS condensates (Figure 2b). We also compared the CH (protein):OH (water) ratio from the high wavenumber region of the spectra (2800–4000 cm^−1^) and found a similar twofold increase in the protein:water ratio for KTS compared to KUTS (Figure S7, Supporting Information).

Kato et al. reported that mutation of tyrosine residues in FUS LC (2–214) progressively decreased formation of hydrogels and RNA granules.^[^ ^5a,c^ ^]^ Since tyrosine‐based interactions in the FUS LC are known to be critical for condensate formation,^[^ ^11a^ ^]^ we analyzed the intensity ratio of the tyrosine doublet at 850 and 830 cm^−1^ (*I*_850_/*I*_830_) for KTS and KUTS condensates. These tyrosine vibrations are sensitive to the conformation of the amino acid backbone, state of the phenol hydroxyl group (free, H‐bonded, or ionized), and local environment of the aromatic ring. The *I*_850_/*I*_830_ is a marker of the average tyrosine side chain H‐bonding interaction,^[^ ^24^ ^]^ whether it interacts with other tyrosines or the solvent. The *I*_850_/*I*_830_ ratios were on average similar for the KTS and KUTS condensates (*n* = 4 for both) with numerical values consistent with our previous results for FUS LC in canonical liquid droplets and dissolved in solution.^[^ ^9^ ^]^ However, the KUTS samples showed a *I*_850_/*I*_830_ histogram skewed to larger values (Figure 2c; see Figure S8, Supporting Information, for histograms from individual condensates) compared to the KTS samples. The higher values of the tyrosine doublet ratio in KUTS indicate that the tyrosines were more exposed toward the solvent than in KTS condensates. The spatial distribution of the *I*_850_/*I*_830_ values qualitatively showed a speckly pattern in both the KUTS and KTS condensates (Figure 2d).

**Figure 3:**
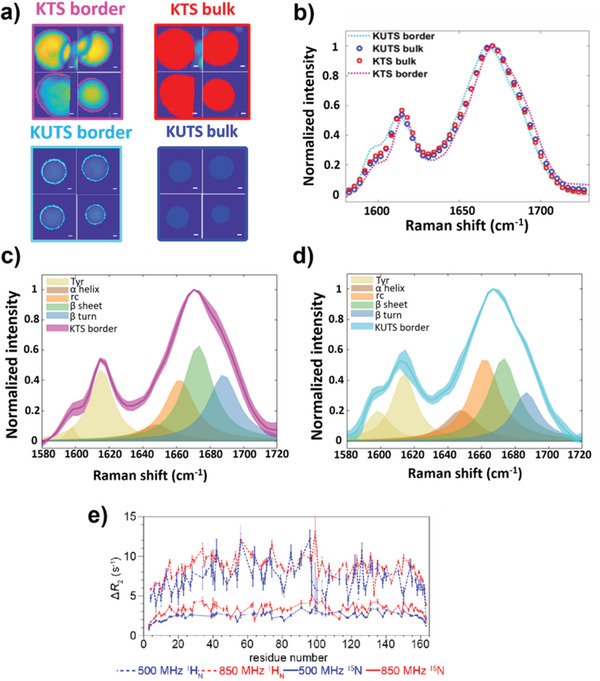
Interfacial protein structure and protein–protein interactions are different in KTS and KUTS condensates. a) Border (magenta) and bulk (red) regions of KTS together with border (cyan) and bulk (blue) regions of KUTS condensates for four representative condensates. Borders were defined as narrow regions (1–3‐pixel width) on the edges of the samples based on Amide I images. Scale bars are 2 µm. b) Average spectra from the bulk regions, which exclude the border regions, of KTS (red, open circles) and KUTS (blue, open circles) samples are shown to overlay almost entirely. Border spectra of KTS and KUTS spectra are also shown with the same colors from (a). c,d) Comparison of the averaged Amide I spectral fitting of KTS (c) and KUTS (d) interfacial regions shows that KTS condensates were enriched in *β*‐sheets and turns on the border, while KUTS condensates' interfacial regions exhibited more *α*‐helical and random coil secondary structure contributions. All data are pooled from four different samples per category with more than 300 spatial pixels in total. Shaded regions show the S.D. between samples of each category. All microscopy was performed at room temperature. e). Probing interfacial protein–protein interactions at cold droplet interfaces with solution NMR. Δ*R*_2_ is observed in the presence of cold‐formed droplets and is relatively uniform across FUS LC for ^1^H and ^15^N nuclei. ^1^H_N_ Δ*R*_2_ values are shown at two magnetic fields (dotted lines) that are very similar. ^15^N Δ*R*_2_ (solid lines) are lower in value and more field dependent.

We further investigated the secondary structural changes between KTS and KUTS condensates using the Amide I band (1600–1700 cm^−1^), which reflects different secondary structure populations through changes in spectral shape. The Amide I vibration is typically broad for FUS LC,^[^ ^9^ ^]^ as the protein is intrinsically disordered and takes on no definitive secondary structure. To investigate the interfacial regions of the KTS and KUTS condensates, we defined a narrow region along the edges of the samples as border regions (**Figure** **3**a). Then, we computed the average normalized spectrum of the border and nonborder (bulk) regions in the KTS and KUTS condensates (Figure 3b; see Figures S9 and S10, Supporting Information, for individual condensate BCARS data). The average Amide I spectra from bulk KUTS and KTS condensates were identical, showing that both types of condensates share the same average secondary structure. In contrast, Figure 3b shows that the averaged border spectra from the Amide I peak shifted to lower or higher Raman shift in KUTS (cyan) or KTS (blue), respectively. Based on peak assignments,^[^ ^25^ ^]^ this shift can be attributed to the relative change of the *α*‐helical, coil, *β*‐sheet, and turn contributions. Spectral decomposition of the Amide I spectra showed an increase of *β*‐sheet and turn contributions for the KTS border compared to the bulk secondary structure (Figure 3c,d). These data show that KTS and KUTS condensates exhibited largely the same secondary structure except at their interfaces, which is consistent with the differences in coalescence, hydrophobicity, and permeability of the interfaces observed above.

Based on the BCARS data showing that FUS LC is more concentrated in and had a distinct protein structure at the interface of KTS condensates compared to KUTS condensates, we asked how cold temperature would affect the molecular interactions between dissolved and phase‐separated FUS LC. We used solution NMR spectroscopy as a method to observe the interaction between free monomeric FUS LC and droplets formed at room temperature and immediately cooled (to 4°C)—similar to KTS condensates. The large size and reduced motions in the cold‐formed droplets precluded characterization of the proteins within the droplet by traditional NMR approaches. However, when something small (a monomer of FUS LC) binds and unbinds a large, slow‐moving object, such as cold‐formed FUS LC droplets, it is ideal for dark‐state exchange saturation transfer. We have previously used this technique to observe the binding of monomeric amyloid *β* both to amyloid protofibrils and to the GroEL chaperonin.^[^ ^26^ ^]^

As in our previous studies, we observed large enhancements in values of the transverse nuclear spin relaxation rate constant, which we term Δ*R*_2_, in the presence of the “dark state”—FUS LC cooled droplets. Δ*R*_2_ is the difference in *R*_2_ of the cold‐formed droplet compared to a droplet‐free low concentration reference (50 *μ* m FUS LC) at identical solution (and temperature) conditions.^[^ ^5b^ ^]^ We found elevated Δ*R*_2_ values for all residues across the entire LC domain (Figure 3e), which strongly suggests that Δ*R*_2_ arises due to kinetic interaction of monomers with the high molecular weight droplets. We note that Δ*R*_2_ was higher for the central region than near the termini, suggesting that the terminal regions move comparatively faster (by exhibiting slower relaxation) when bound to the cold droplet. Consistent with this kinetic binding interpretation, Δ*R*_2_ for amide hydrogen positions, ^1^H_N_ Δ*R*_2_, were approximately equal at two magnetic fields (850 vs 500 MHz ^1^H frequency) (Figure 3e, dotted lines), suggesting that the apparent first order rate constant for binding of FUS LC molecules in the bulk solution to cooled FUS LC droplets, *k*_on_^app^, was approximately equal to the maximum in ^1^H_N_ Δ*R*_2_ = 10 s^−1^.^[^ ^27^ ^]^ In other words, the ^1^H_N_ relaxation rates in the bound state were so fast (at both 850 and 500 MHz) that the magnetization of monomers binding to the droplet was entirely lost before the monomer could unbind to rejoin the dissolved monomer pool. Interestingly, the Δ*R*_2_ for the ^15^N backbone positions, ^15^N Δ*R*_2_, were less than ^1^H_N_ (maximum values of 5 vs 10 s^−1^, respectively) (Figure 3e, solid lines), suggesting that the monomer unbinds before the slower relaxing transverse ^15^N magnetization is completely quenched (i.e., the unbinding rate to rejoin the pool of monomers is faster than the transverse relaxation rate in this bound state).^[^ ^26c^ ^]^ Indeed, the values of ^15^N Δ*R*_2_ were systematically lower at lower magnetic field (500 vs 850 MHz ^1^H frequency) consistent with lower bound ^15^N *R*_2_ values expected at lower magnetic fields (Figure 3e, solid lines). Because the concentration of the dissolved monomers remains constant over several days, this (pseudo)equilibrium requires that some FUS LC must also unbind from the droplet. That is to say, there was no static build‐up of these monomers on the cold FUS LC droplet during these experiments. We note that Δ*R*_2_ for ^15^N room temperature‐formed FUS LC droplets was less than 0.5 s^–1^ at all residues for similar concentrations (Figure S11, Supporting Information). Taken together, our NMR relaxation experiments demonstrate that free monomers of FUS LC interact with the cold‐incubated droplets more strongly compared to room temperature FUS LC droplets, which suggests that cold‐incubated droplets exhibit a larger stickiness compared to room temperature droplets and FUS LC in solution.

### 2.3 Kinetic Trapping of FUS LC Creates Condensates with Stronger Protein–Protein Interactions and Reduced Multiscale Mobility

**Figure 4:**
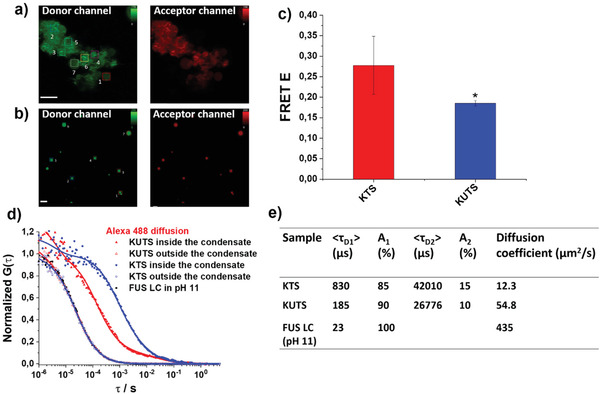
FUS LC chain–chain interactions and molecular mobility are distinct in KTS and KUTS condensates. a,b) Donor (Cy3, green) and acceptor (Cy5, red) channels of KTS (a) and KUTS (b) FUS LC condensates measured for FRET AB measurements. Boxed regions show the regions of interest (ROIs) that were selected to calculate the FRET efficiency, *E* = (Donor Int_post_ − Donor Int_pre_)/Donor Int_post_, of each sample. Scale bar is 10 µm. c) Graph shows *E* for KUTS (red) and KTS (blue) from different *n* = 7 ROIs calculated in a point‐by‐point manner. Error bars are S.D., and * indicates statistically significant differences (*p* < 0.05). d) Normalized fluorescence correlation *G*(*τ*) curves of Alexa 488 diffusion in KTS and KUTS, respectively. All curves were fit to Equation S1, Supporting Information. e) Table showing average correlation decay times, percent contribution, and diffusion coefficient of Alexa 488 in different samples. The diffusion coefficients were calculated from the decay time with larger contribution to the signal.

Following the NMR experiments showing stronger interaction of soluble FUS LC to cold‐formed condensates and BCARS showing different interfacial protein structure for KUTS and KTS condensates, we further explored the protein–protein interaction and size‐dependent mobility. We employed Förster resonance energy transfer acceptor photobleaching (FRET AB), fluorescence correlation spectroscopy (FCS), fluorescence recovery after photobleaching (FRAP), and particle tracking microrheology (PTM) experiments to probe interaction and mechanics in the condensates. In FRET AB experiments, we used Cy3‐FUS LC and Cy5‐FUS LC as a FRET pair at a total concentration of less than 0.03 mol% of unlabeled FUS LC ([Cy3‐FUS LC]:[Cy5‐FUS LC] = 1:2), to form KTS and KUTS condensates. For the FRET AB assay, we measured changes in donor (Cy3) fluorescence intensity in the KTS/KUTS condensates pre‐ and post‐acceptor (Cy5) bleaching to calculate the FRET efficiency, *E*. **Figure** **4**a,b shows representative donor (green) and acceptor (red) images for KTS and KUTS using spectral confocal microscopy. As expected, the donor and acceptor fluorescence looked identical as both the Cy3 and Cy5‐FUS LC were incorporated into the condensates. Consistent with KTS being a more crowded condensate, we found a statistically significant increase in *E* compared to KUTS (Figure 4c). A higher *E* value indicates increased protein–protein interaction and thus increased resonance energy transfer between the two labels. Based on the average *E* = 0.28 in KTS and 0.18 in KUTS and using published Förster distances for the donor–acceptor pair, we calculate an average spacing between dyes of 7 and 7.7 nm in KTS and KUTS condensates, respectively. The raw FRET histograms from the regions of interest in Figure 4a,b are shown in Figures S12 and S13, Supporting Information, which show the substantially larger heterogeneity in KTS condensates compared to KUTS condensates. These data show that, on average, kinetic trapping of FUS LC resulted in a more locally crowded protein environment, leading to a more pronounced FRET between Cy3 and Cy5 for KTS compared to KUTS condensates.

To probe the impact of the kinetic trapping on local concentration/molecular mobility within KTS and KUTS condensates, FCS was employed using a hydrolyzed Alexa 488 dye—an NHS ester conjugate left at room temperature in pH 7.4 phosphate buffer overnight—as a fluorescent molecular tracer. The single‐molecule sensitivity and high spatial resolution of FCS are ideal to probe the molecular scale physical mobility in situ, allowing extraction of various parameters such as diffusion coefficients, molecular concentration, and nanoviscosity.^[^ ^28^ ^]^ FCS has been routinely used to study the kinetics of inter‐ and intra‐molecular interactions of various BCs and lipid membranes, among other systems, to quantify nanoscopic molecular mobility.^[^ ^28^, ^29^ ^]^ The decay times of representative autocorrelation curves (Figure 4d) reflect the average residence time of the diffusing dye in the FCS probing volume and thus are a measure for the viscosity of the environment. The autocorrelation curve measured for Alexa 488 in a KTS condensate (filled blue symbols) was shifted to longer lag times compared to that measured in a KUTS condensate (filled red symbols). The longer lag time means slower diffusion of the Alexa 488 tracers in KTS, which reflects a higher cross‐link and/or protein density. These observations suggest that a denser, more viscous environment at the molecular scale was induced in the KTS condensates, which is consistent with CARS data showing twofold more protein concentration in KTS compared to KUTS.

We fit the measured autocorrelation curves with Equation S1, Supporting Information, (see Materials and Methods in the Supporting Information for details) to calculate the corresponding decay times and diffusion coefficients of the Alexa 488 tracers (Figure 4e). We note that correlation curves from both KTS and KUTS condensates required two decay components (*m* = 2 in Equation S1, Supporting Information) to fit the experimental curves. The faster decay component, *τ*_D1_, contributed more than 85% to the total fit compared to the second component (*τ*_D2_), which contributed less than 15% in the fit. Moreover, decay times of order 10 ms (as *τ*_D2_) are believed to originate from physical adsorption/desorption of the tracer to the protein network.^[^ ^30^ ^]^ Therefore, we calculated diffusion coefficients based on *τ*_D1_. We found that Alexa 488 in KTS (*D* = 12.3 µm^2^ s^−1^) diffuses ≈4.5 times slower than in the KUTS (*D* = 54.8 µm^2^ s^−1^) condensates.

We also measured the diffusion of the Alexa 488 tracers in the presence of dissolved 200 µm FUS LC. The corresponding autocorrelation curve (Figure 4d, black) was almost identical to those recorded outside of the KUTS and KTS condensates (open blue and red symbols). All three curves could be fit with a single component diffusion model (*m* = 1 in Equation S1, Supporting Information), which produced a decay time (*τ*_D_) of 23 µs (*D* = 435 µm^2^ s^−1^). This decay time is identical to the one obtained in calibration measurements of Alexa 488 diffusing in pure water and indicates that the tracer molecules are not affected by the dissolved FUS LC at the studied protein concentrations. Similar results were obtained from FCS measurements of Atto 425 (Figure S14 and Table S1, Supporting Information), an uncharged dye compared to Alexa 488, which is negatively charged.

**Figure 5:**
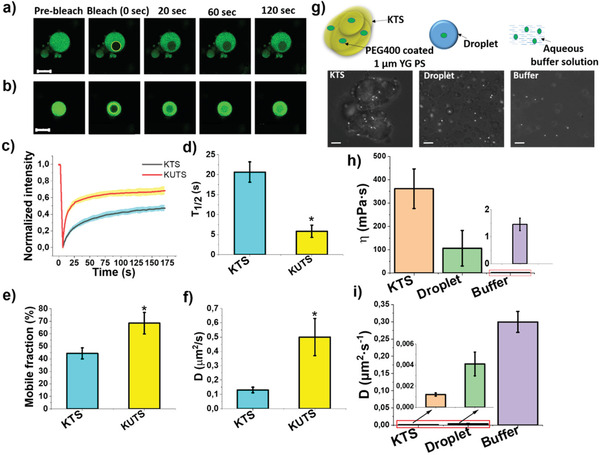
Protein and macroscopic mobility are hindered in KTS condensates. a,b) Representative images from FRAP experiments of 0.01 mol % Cy3‐labeled FUS LC‐doped KTS and KUTS (times indicate seconds post bleach). The yellow circle highlights the punctum that underwent targeted bleaching. Scale bar is 10 µm. c) Normalized fluorescence intensity of KTS and KUTS over time with thick lines indicating averages over ten samples and colored shaded areas showing the S.D. d–f) Bar graphs showing half‐time fluorescence recovery (*T*_1/2_) (d), mobile fraction (%) distribution (e), and diffusion coefficient values (f) in various samples. Error bars are S.D. g) Cartoon representation of precoated PEG 400 yellow–green emitting polystyrene beads encapsulated in KTS, liquid condensates, and buffer. Brightfield images of KTS condensates and canonical FUS LC droplets containing microspheres. Scale bar is 10 µm. h,i) Bar graphs showing dynamic viscosity (h) and diffusion coefficient (i) for all populations of beads in different FUS LC samples. Error bars are S.D. (more than 20 beads per sample) in all panels, and statistically significant differences (*p* < 0.05) compared to KTS are indicated by “*”.

While FCS is informative for small molecule tracers, FRAP is a frequently used technique to determine the local mobility of proteins and liquid‐like nature in BCs.^[^ ^31^ ^]^ To determine the apparent diffusion coefficient of FUS LC in KTS and KUTS condensates using FRAP, we bleached small portions of the structures (≈3.5 µm diameter circles) containing 0.01 mol% of Cy3‐labeled FUS LC (**Figure** **5**a,b). The half‐time of recovery (*T*_1/2_) and mobile fraction of the labeled FUS LC inside KTS and KUTS condensates were determined using time‐lapse imaging data. Due to strong protein–protein interaction and low concentration of protein in the dilute phase, the recovery reached a plateau below 100% recovery (Figure 5c,d). As such, *T*_1/2_ was defined by the time that it takes for the fluorophore to reach half of its maximal recovery intensity. The rate of recovery depends on the local density (viscosity) as well as the strength of the protein–protein interactions in the network.^[^ ^32^ ^]^ The estimated *T*_1/2_ values for KTS and KUTS were 20.7 ± 2.54 and 5.8 ± 1.55 s (*n* = 7 in each case), respectively (Figure 5d), and statistically independent (*p* < 0.05). Importantly, the recovery time of ≈20 s for KTS condensates shows that these condensates still contained dynamic FUS LC molecules; in other words, the KTS condensates are not solid. Additionally, the apparent diffusion coefficients for FUS LC in KTS and KUTS condensates derived from FRAP experiments (*D* = 0.88 × *a* ^2^/(4 × *T*_1/2_), where *a* is the radius of the photobleached region) were 0.13 ± 0.02 and 0.46 ± 0.13 µm^2^ s^−1^, respectively (Figure 5f).^[^ ^33^ ^]^ The apparent diffusion coefficient in KUTS condensates was almost ≈3.5‐fold higher than that of KTS, quantitatively consistent with the results from FCS. These results show that the protein network in KTS FUS LC condensates slows the translational diffusion of FUS LC more significantly than in KUTS condensates. The faster protein diffusivity in KUTS thus indicates a less dense protein network and/or weaker interactions, consistent with results from FRET AB, FCS, and BCARS Amide I quantification. The mobile fraction of Cy3‐labeled FUS LC in KTS was significantly below that in KUTS being 44.3% ± 4.42% and 68.5% ± 8.6%, respectively (Figure 5e), in agreement with our measurements showing that KTS condensates have a more restrictive protein network than KUTS condensates. We note that the apparent diffusion coefficient in KUTS condensates were slightly lower relative to canonical FUS LC droplets formed by direct incubation at room temperature (*D* = 0.73 ± 0.175 µm^2^ s^−1^, Figure S15, Supporting Information).

As an additional measurement of the mobility changes at larger length scales in the KTS/KUTS condensates, we used PTM with micrometer particles to probe the macroscopic viscosity. These measurements are complementary to the FCS and FRAP measurements, which probe molecular mobility at the sub‐nm and protein length scales, respectively. PTM‐based measurements are a convenient method to determine the viscoelastic nature of soft materials from the Brownian motion of tracer beads.^[^ ^12^, ^34^ ^]^ This technique uses the Stokes–Einstein relation to calculate the dynamic viscosity according to: *η* = *k*_B_ *T*/6*πRD*, where *R* is a sphere radius (0.5 µm), *k*_B_ is the Boltzmann's constant, and *T* is the temperature of the system from a diffusion constant *D* measured by tracking particle motion. We followed a similar protocol used by Brangwynne and colleagues by using PEG‐coated fluorescent polystyrene beads (1.00 µm) for encapsulation into KTS condensates (Figure 5g).

PEG‐passivation of beads was used to prevent nonspecific binding (see Materials and Methods in the Supporting Information). The thermal trigger for KUTS formation did not result in bead encapsulation, so we used canonical FUS droplets formed directly at room temperature as their dynamics in FRAP experiments were not drastically different than KUTS condensates. The dynamic viscosity and diffusion coefficient values for liquid KTS condensates and canonical FUS droplets were determined, and the values were compared with the viscosity of beads in 200 µm FUS LC dissolved in CAPS buffer. The estimated dynamic viscosity for KUTS and KTS condensates was 106 ± 76 and 361 ± 85 mPa s^−1^, respectively, (Figure 5h), and the calculated diffusion coefficients for the tracer in droplets and KTS were 4.11 × 10^−3^ ± 1.13 × 10^−3^ and 1.21 × 10^−3^ ± 1.5 × 10^−4^ µm^2^ s^−1^, respectively (Figure 5i). For tracer particles dispersed in CAPS buffer containing dissolved FUS LC, we calculated a viscosity of 1.5 mPa s^−1^, which is essentially the viscosity of water, as expected. The 3.4‐fold increase in dynamic viscosity and corresponding 3.4‐fold decrease in diffusion coefficient of 1 µm microspheres in KTS condensates compared to KUTS condensates is quantitatively consistent with the FRAP data showing a 3.5‐fold increase in viscosity for FUS LC KTS condensates. The increased diffusion coefficient of 1 µm microspheres, faster FRAP recovery, and reduced FCS correlation times in the KUTS condensates compared to KTS condensates shows that the FUS LC protein network was less restrictive in the former.

### 2.4 FUS LC Condensates Show Temperature‐Dependent Amino Acid Radial Distributions

With our experiments showing the existence of two demixed liquid states from the same protein with such distinct physical chemical properties, we hypothesized that different molecular organization could be responsible for stabilizing the different condensates of FUS LC. To test this hypothesis, we performed coarse‐grained (CG) molecular dynamics simulations to quantify the amino acid distribution in the condensate at different temperatures. To simulate FUS LC phase separation we used the Kim–Hummer CG model,^[^ ^35^ ^]^ which uses a one bead per amino acid level of coarse graining with nonbonded interactions between the amino acids informed via the Miyazawa Jernigan statistical contact potential (Materials and Methods in the Supporting Information and Equations S2–S5, Supporting Information).^[^ ^36^ ^]^ Other interactions between amino acids include a harmonic potential representing the bonded interactions between consecutive amino acids on the protein chain and a Coulombic term with Debye–Hückel screening representing the electrostatic interactions between charged residues at a certain salt concentration.^[^ ^35^ ^]^ As configured, our simulations do not predict chain structure but rather amino acid and chain radial distribution.

**Figure 6:**
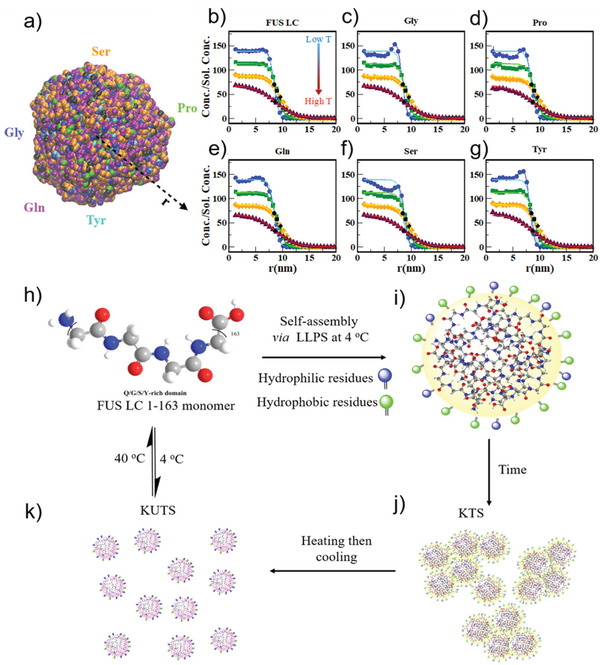
Simulations showing temperature‐dependent changes in amino acid distribution in FUS LC condensates and proposed mechanism of formation of kinetically trapped and untrapped FUS LC condensates. a) FUS LC condensate snapshot from a CG‐MD simulation of 100 chains at 100 K. CG beads are colored according to specific amino acid types (Ser, Pro, Tyr, Gln, and Gly) while the beads representing other amino acids are colored black. Concentration profiles of b) FUS LC and individual amino acids c) Gly, d) Pro, e) Gln, f) Ser, and g) Tyr at 100 K (blue, circle markers), 150 K (green, square markers), 200 K (orange, diamond markers), 230 K (maroon, triangle markers) simulation temperatures are normalized by their solution concentrations in the simulation. The solid lines in (b–g) are fits of the normalized concentration profile to a sigmoidal function and the black markers represent the inflection points for the fits to the concentration profiles with matching sybmols. h) Condensation of QGSY‐rich FUS LC (residues 1–163) via cold formation leads to i) slightly more hydrophobic residues (green circles) on the interface and j) formation of KTS condensates that show arrested coalescence. k) Annealing of KTS allows protein reconfiguration to permit additional hydrophilic residues (blue circles) to inhabit the interface and changes the bulk protein network density in KUTS condensates.

We simulated 100 chains of the FUS LC sequence in a cubic box at a range of temperatures and at ≈100 mm salt concentration using a procedure described in previous work.^[^ ^35^ ^]^ At a very low temperature (100 K), the FUS LC system forms a dense condensate shown in (**Figure** **6**a) with a uniform distribution of FUS LC chains in the dense phase along the radial direction (Figure 6b, blue circles). We fit a sigmoid function to the normalized concentration profile of FUS LC chains computed by taking the ratio of the protein concentration at each location within the condensate and the protein concentration in the (noncondensate) solution for different temperatures (shown as solid lines in Figure 6b). This quantity allowed us to locate the interface from the inflection point of each sigmoidal fit (black markers used for different temperatures in Figure 6b). The normalized FUS LC concentration in the condensates increased with decreasing temperature, as reflected in our BCARS results and also consistent with our FCS, FRET, and PTM data. These simulations also allowed us to quantify the distribution of amino acids within the condensates as a function of temperature. Like the FUS LC concentration profiles, prominent amino acids (Gly, Pro, Gln, Ser, and Tyr) showed sigmoid radial distributions at most temperatures. However, looking closely at the amino acid distributions at the lowest temperature (Figure 6c–g, blue lines), we observe clear deviations with respect to the sigmoidal fit (Figure 6b). Deviations from the sigmoidal fits are less visible for simulations of higher temperature systems (Figure 6c–g, green, orange, and red markers/lines).

The simulation results show that the temperature at which a condensate forms influences the amino acid distribution within the condensate and at the interface. The reconfiguration of amino acids at different temperatures could in turn result in different secondary structure of FUS LC at the interface of KTS condensates compared to KUTS condensates, as we found experimentally. Therefore, we hypothesize that the temperature‐dependent morphological transition of FUS LC BCs stems from changes in the molecular configuration of proteins in the condensate. Indeed, a low temperature entails a deep quench in the two‐phase coexistence region, where the thermodynamic driving force for phase separation is substantial. Consequently, the typical rate of condensation may well surpass that of segmental reorganization and relaxation, especially in the concentrated phase.

Our results show that FUS LC can form multiple distinct liquid condensates with very different physical chemical properties simply by modifying the temperature during condensate formation. This finding is similar to that from Murakami et al. where they found reversible gelation of solutions of FUS 2–214 (which also contains an additional 51 amino acids on the C‐terminus). However, the KTS and KUTS condensates were both liquid‐like, with neither sample exhibiting gel‐like properties throughout the sample, having viscosities at least tenfold below FUS gels.^[^ ^10b^ ^]^ In another study, , Boeynaems et al. reported a metastable state (similar to KTS) that was kinetically arrested by formation of polyelectrolyte coacervation between proline–arginine dipeptide repeats together with polyA and polyU RNA.^[^ ^19b^ ^]^ In addition, metastable disordered states have been further observed for protein solutions that form a “dense amorphous phase” from which crystal nucleation and growth take place according to a two‐step process.^[^ ^15^ ^]^ In this case, a fully developed interface with the dilute phase forms, giving rise to a truly demixed disordered state that is metastable relative to the crystalline phase.

Our work demonstrates the precedent for finding metastable states in pure disordered protein BCs that remain liquid and further explores the multiscale structural and dynamical properties in trapped versus untrapped BCs. The characterization methods we applied demonstrate that a unique interfacial molecular structure exists for the KTS condensates, which show arrested coalescence. We note that the formation of oligomers could, in principle, precede formation of the KTS condensates, but our NMR and BCARS offered no evidence of structured oligomers; future investigations of the KTS formation pathway are ongoing. Rather, we found that the protein structure in the bulk KTS/KUTS condensates were indistinguishable even though the tyrosines in KTS showed increased H‐bonding to other tyrosines while tyrosines in KUTS interacted more with water. Interestingly, using a thermal switch allowed us to convert the KTS condensates to the KUTS liquid droplets, but the reverse was not possible.

We propose the following scheme for KTS and KUTS condensate formation of FUS LC condensates based on our experimental results and simulations (Figure 6h–k). Cold‐forming FUS LC condensates traps a unique amino acid organization in condensates, particularly at the interface, resulting in increased hydrophobicity and increased *β*‐sheet structure at the interface of KTS condensates (Figure 6i). Exposing hydrophobic residues toward the aqueous buffer during kinetic trapping leads to formation of KTS agglomerates rather than fused condensates (Figure 6j). Thermal annealing of KTS condensates allows FUS LC proteins to escape the kinetic trap, reconfigure, and find a more favorable thermodynamic state, which turns out to be a less viscous liquid state (Figure 6k). Such temperature‐dependent balance of transient assembly during LLPS provides an opportunity to trigger mixing and control condensate composition in a repeatable way using a thermal switch.

## 3 Conclusion

We have shown that cold formation of FUS phase‐separated assemblies created metastable BCs that are kinetically trapped, which exhibit unique liquid‐like properties compared to canonical FUS liquid droplets. This finding highlights the need for additional granularity within the broad thermodynamic classifications typically used for BC characterization, that is, not all liquid condensates are the same—even from the same protein with no biochemical modifications. Our findings have implications regarding the diversity of potential states of FUS in stress granules in cells, which Patel et al.^[^ ^1b^ ^]^ showed to include liquid‐like and solid‐like aggregated states. From an application perspective, triggering structural transitions of FUS LC assembly by thermal shock provides insight on an important challenge in the BC field of transient control of condensate stability and composition. Recent applications of condensates in drug delivery or synthetic organelle formation have mostly relied on using IDRs to control condensate composition. The thermal method described here is similar to the Optodroplet concept by Brangwynne and colleagues in that an external stimulus (light, in their case) can be used to trigger condensate reorganization. We anticipate that a broader array of stimuli‐responsive structural transitions that allow for on‐demand mixing and formation of condensates will propel application of BCs as drug delivery vehicles that convert from trapped states to dynamic liquids at physiological temperature and as coacervates for potential industrial applications.^[^ ^31^, ^37^ ^]^

## Conflict of Interest

The authors declare no conflict of interest.

## Author Contributions

S.C., Y.K., and S.H.P. designed and conceived the study. S.C. and K.K. performed and analyzed the FRET and FCS measurements. S.C. and Y.K. performed and analyzed the BCARS measurements. S.C. and M.B. performed and analyzed the PTM studies. A.C.M., K.A.B., and N.L.F. designed, performed, and analyzed the NMR spectroscopy. R.M.R. and J.M. designed and performed simulation experiments and analyzed the resulting data. S.C., J.J.M., and S.H.P. wrote the manuscript with comments from all authors.

## Supporting information

Supporting Information

Supplemental Movie 1

## Data Availability

The data that support the findings of this study are available from the corresponding author upon reasonable request.
