# Supplementary material for: Reversible Kinetic Trapping of FUS Biomolecular Condensates
Source: Adv Sci (Weinh). 2021 Dec 4;9(4):2104247. doi: 10.1002/advs.202104247 (PMC8811844; doi:10.1002/advs.202104247)
Supplement: Supplementary file 1 — Supporting Information [file ADVS-9-2104247-s002.pdf]

ADVANCED SCIENCE
Open Access

Supporting Information

for *Adv. Sci.,* DOI: 10.1002/advs.202104247

Reversible kinetic trapping of FUS biomolecular condensates

Sayantan Chatterjee, Yelena Kan, Mateusz Brzezinski, Kaloian Koynov, Roshan Mammen Regy, Anastasia C. Murthy, Kathleen A. Burke, Jasper J. Michels, Jeetain Mittal, Nicolas L. Fawzi, Sapun H. Parekh

Supplementary Information

Reversible kinetic trapping of FUS biomolecular condensates

Sayantan Chatterjee[1,2], Yelena Kan[1,2,3], Mateusz Brzezinski[1,2], Kaloian Koynov[2], Roshan Mammen Regy[4], Anastasia C. Murthy[5], Kathleen A. Burke[5], Jasper J. Michels[2], Jeetain Mittal[4], Nicolas L. Fawzi[5], Sapun H. Parekh[1,2]

[1] Department of Biomedical Engineering, University of Texas at Austin, 107 W. Dean Keeton Rd., Austin, TX USA 78712

[2] Max Planck Institute for Polymer Research, Ackermannweg 10, Mainz, DE 55128

[3] LUT School of Engineering Science, LUT University, Yliopistonkatu 34, 53850 Lappeenranta, Finland

[4] Artie McFerrin Department of Chemical Engineering, Texas A&M University, 200 Jack E. Brown Engineering Building, College Station, TX 77843

[5] Department of Molecular Biology, Cell Biology, and Biochemistry, Brown University, 70 Ship Street, Providence, RI USA 02912

Corresponding author: Sapun H. Parekh, sparekh@utexas.edu

Materials and Methods

Protein expression and purification

The FUS LC Plasmid used for expression is available from AddGene (#127192). Human FUS LC (residues 1-163) was expressed in chemically competent *Escherichia coli* BL21 bacteria. Cells were grown in LB medium containing kanamycin shaking at 37 °C until reached an OD_{600} between 0.6-1. Expression was induced by addition of isopropyl-beta-D-thiogalactoside (IPTG) to a100 final concentration of 1 mM. After 4 h of IPTG induction, cells were centrifuged at 4500g for 10 min at 4 °C. The resultant pellet was stored at -20 °C until further purification. For cell lysis, the pellet was redispersed in 20 mL of phosphate buffer at pH 7.4 (containing 300 mM of NaCl, 10 mM of imidazole) and tip sonicated (Fisher Brand, model CL-18) in an ice bath (30s on-off cycles for ten times using 80% amplitude). Lysed cells were sedimented by centrifugation at 18500g for 1 h at 4 °C, and the obtained pellet was redispersed in solubilizing buffer (phosphate buffer pH 7.4 containing 300 mM of NaCl, 10

mM of imidazole and 8 M of urea) and stirred overnight at 4 °C to solubilize inclusion bodies, which contain FUS LC. After inclusion body solubilization, the sample was tip sonicated once more to remove any residual viscosity, centrifuged again at 18500g for 1 h at 4 °C, and the supernatant was loaded onto Ni-NTA agarose columns. After binding of Histidine-tag (His-tag) on the protein to the Ni-NTA resin for 1 h at 4 °C, the unbound proteins and cell fragments were washed several times with phosphate buffer (pH 7.4) containing 300 mM of NaCl, 5 mM of imidazole. FUS LC was subsequently eluted in steps by running through the washing buffer with increasing imidazole concentration (10, 20, 40, 100mM). The collected fractions were pooled, and the purified protein was cleaved by it diluting in solubilizing buffer with addition of Tobacco Etch Virus protease with a 1:20 mass ratio. Cleaved purified protein was purified by another Ni-NTA column, which binds to protease and the cleaved His-tag, so the flow through yields a His-tag-free product as confirmed by mass spectrometry. The cleaved product was buffer exchanged to 20 mM CAPS (pH 11) overnight, concentrated using 3 kDa Amicon filter to ~ 1 mM, and stored at -80 °C.

KTS and KUTS formation

Protein-rich kinetically trapped (KTS) condensates of FUS LC (residues 1-163) were formed by dilution of the protein from a stock solution (in 20 mM of CAPS, pH 11) into 20 mM of cold (4 °C) phosphate buffer containing 150 mM of NaCl. The final pH of the resultant mixture was adjusted to 7.4 and allowed to incubate overnight at 4 °C unless otherwise stated. Formation of KTS condensates of FUS LC was tested at initial concentrations ranging from 5-200 μM and was generally 200 μM unless otherwise stated. The formation of kinetically untrapped (KUTS) condensates from KTS was achieved by heating (15 min at 40 °C using an Eppendorf, Thermomixer) and followed by cooling by natural convection to room temperature or 4 °C. All imaging was performed at room temperature. Bright field imaging of condensates was done on an Olympus IX70 microscope by drop casting the KTS and KUTS solution on a #1 glass coverslip (20 mm× 20 mm) with no additional preparation.

CARS microspectroscopy

For CARS measurements, KTS and KUTS samples were placed in two channels created by double sided tape strips between glass coverslip and glass slide. Buffer solution was placed in third channel for reference measurement. For the Raman fingerprint measurements, we used a broadband coherent anti-Stokes Raman scattering (BCARS) microscope. This home-built BCARS setup has been described extensively elsewhere.[1] Briefly, the pump/probe and Stokes pulses are generated in a dual-output, sub-nanosecond laser source (CARS-SM-30, Leukos), spatially and temporally overlapped at the sample plane of inverted microscope (Eclipse Ti-U, Nikon), and tightly focused on the sample with a 0.85 NA air objective (LCPlan N, Olympus). The BCARS signal is filtered from the excitation pulses and focused onto the slit of a spectrograph (Shamrock 303i, Andor), which disperses the spectral components on a cooled CCD camera (Newport DU920P-BR-DD, Andor). Samples were mounted with the cover slip facing the objective. The samples were then raster scanned by moving a piezo stage (Nano-PDQ 375

HS, Mad City Labs), and the data acquisition was controlled via interface software in LabView 2015 (National Instruments). Collected hyperspectral data were afterwards processed in IgorPro (WaveMetrics). The Raman-like spectra were phase-retrieved *via* a modified Kramers-Kronig transform,[2] and background phase removal with Savitzky-Golay filter with 2nd order polynomial and window size ~400 cm^{-1}.

Förster resonance energy transfer (FRET) acceptor photobleaching (AB) assay

1 mg of Cy3-NHS ester or Cy5-NHS ester (Lumiprobe) dye was dissolved in 100 µL of dry DMF and divided into aliquots of 40 µL. FUS LC (50 µM) in HEPES buffer (50 mM, pH 8.0) was added to 40 µL of dye stock solution in DMF was added. After addition, the reaction mixture was incubated overnight at 4 ^{o}C. Purification of dye labeled FUS LC was carried out by repeating buffer exchanged to CAPS (pH 11) at 4 ^{o}C, and then concentrated using 3 kDa Amicon filter and stored at -80 °C. For FRET AB microscopy experiments, 0. 1 mol% of Cy3 labeled FUS LC and 0.2 mol% of Cy5 labeled FUS LC were doped into 200 µM unlabeled FUS LC KTS condensates. FRET experiments were performed on a Leica SP5 confocal microscope with a 63× oil NA objective. In "Spectral Mode," the excitation wavelengths for Cy3- and Cy5-labeled FUS LC were 551 nm and 640 nm, respectively. The calculation of FRET efficiency (E) from these images can be done using LAS X Core* software. To calculate E, measure the donor intensity in both the pre- (I_{pre}) and post-bleach (I_{post}) images within the ROI using following eqn: $E= (I_{post}-I_{pre})/I_{post}$.

Fluorescence correlation spectroscopy (FCS)

FCS experiments were performed on a commercial setup built on an LSM 880 (Carl Zeiss, Jena, Germany). Excitation was done with the 488 nm or the 458 nm lines (for Alexa 488 and Atto 425 respectively) of an Argon laser focused into the studied samples through a C-Apochromat 40×/1.2 W water immersion objective (Carl Zeiss, Jena, Germany). The emission light was collected with the same objective and after passing through a confocal pinhole, directed to a spectral detection unit (Quasar, Carl Zeiss) in which a detection range 500–550 nm (Alexa 488) or 470-570 nm (Atto 425) was selected. Eight-well polystyrene chambered cover glasses (Nunc™ Lab-Tek™, Thermo Fisher Scientific, Waltham, MA, USA) were used as sample cells for the studied solutions. CLSM images were recorded first to identify the location of the protein condensates. Then the confocal volume was positioned in a center of a condensate and a series of 10 FCS measurements with a total duration 5 min were performed. The time-dependent fluctuations of the fluorescent intensity $\delta I(\tau)$ were recorded and analyzed by an autocorrelation function $G(\tau) = 1 + \delta I(\tau) \cdot \delta I(\tau + \tau) >/<I(\tau)>^2$. The obtained in this way experimental autocorrelation curves were fitted with the analytical expression the autocorrelation function of an ensemble of *m* different types of freely diffusing fluorescence species:

$$G(\tau) = 1 + \left[1 + \frac{f_T}{1-f_T} e^{-\tau/\tau_T}\right] \frac{1}{N} \sum_{i=1}^{m} \frac{f_i}{\left[1+\frac{\tau}{\tau_{Di}}\right]\sqrt{1+\frac{\tau}{S^2 \tau_{Di}}}} \quad \text{(S1)}$$

Here, N is the average number of diffusing fluorescence species in the observation volume, f_T and τ_T are the fraction and the decay time of the triplet state, τ_{Di} is the decay time of the *i-th* type of species, f_i is the fraction of component *i*, and *S* is the so-called structure parameter, $S = z_0/r_0$, where z_0 and r_0 represent the axial and radial dimensions of the confocal volume, respectively. Furthermore, the decay time, τ_{Di}, is related to the respective diffusion coefficient, D_i, through: $\tau_{Di} = \frac{r_0^2}{4D_i}$. The fits yielded the corresponding decay times, and subsequently the diffusion coefficients of the fluorescent species. As the value of r_0 depends strongly on the specific characteristics of the optical setup, a calibration experiments were performed using a fluorescent tracer with known diffusion coefficient, i.e., Alexa Fluor 488 or Atto 425 in water.

Fluorescence recovery after photobleaching (FRAP)

FRAP was performed on a Leica SP5 laser scanning confocal microscope with a 25X, 0.95 NA water immersion objective. 0.01 mol% of Cy3 labeled FUS LC was doped in the sample for each FRAP experiment. The bleach region was defined as a 3.5 µm diameter circle using the 514 nm laser line (ten iterative pulses with full laser power) in the center of condensates, and the resulting intensity change over time was collected with 514 nm laser (with 2% laser intensity) for Cy3-labeled FUS LC in samples. The recovery curve, half-time of recovery, and immobile fraction was calculated using the EasyFRAP tool.[3] The apparent diffusion coefficient was then estimated as $D=0.88*a^2/(4*T_{1/2})$, where a and $T_{1/2}$ were the radius of the photobleached region and half-time of recovery, respectively.

Nuclear magnetic resonance (NMR) spectroscopy

Uniformly ^{15}N-labeled FUS LC was expressed using M9 media with ^{15}N ammonium chloride as the sole nitrogen source. ^{15}N FUS LC was purified for NMR nearly identically as described above, as described previously.[4] NMR samples of 350 µM FUS LC were created by diluting <50 µl of FUS LC in 20mM CAPS pH 11.0 stock in 450 µl 20 mM MES pH 5.0 buffer including 10% ^2H_2O. Final sample pH was 5.4. Monomeric control samples of 50 µM FUS LC were created by adding additional 20 mM CAPS pH 11.0 buffer to ensure identical sample conditions in the 350 µM sample. Concentrations of FUS LC were estimated using the extinction coefficient of 35760 $M^{-1}cm^{-1}$ calculated by ProtParam.[5]

NMR ^{1}H and ^{15}N ΔR_2 and dark-state exchange saturation transfer experiments characterizing the kinetics and residue-specific interactions of soluble FUS LC with cold droplets were performed on 350 µM (droplet containing) and 50 µM (droplet-free reference) samples as described previously after a 6 hr droplet equilibration period at 4 °C after which measured R_2 values were stable for >24 hrs.[6-8]

Specifically, all NMR experiments were recorded at 4 °C using Bruker Avance and Avance III HD NMR spectrometers operating at a ^{1}H frequencies of 500 and 850 MHz, respectively, each equipped with a Bruker TCI z-axis gradient cryogenic probe. Experimental sweep widths and acquisition times (i.e. resolution) and the number of transients were optimized for the necessary resolution, experiment time, and signal-to-noise for each experiment type.

Transverse relaxation of nitrogen nuclei, ^{15}N R_2, was measured using a standard pulse sequence (hsqct2etf3gpsitc3d, Topspin 3.2, Bruker). Interleaved experiments comprised 128*×2048* complex data pairs in the indirect ^{15}N and direct ^{1}H dimensions, respectively, with corresponding acquisition times of 74 ms and 229 ms, sweep width of 20 ppm and 10.5 ppm, centered at 117 ppm and 4.9 ppm, respectively. ^{15}N R_2 experiments had an interscan delay of 2.5 s, a Carr-Purcell-Meiboom-Gill (CPMG) field of 556 Hz, and total R_2 relaxation CMPG loop-lengths of 16.5 ms, 33.1 ms, 82.6 ms, 115.7 ms, 165.3 ms, 181.8 ms, and 264.4 ms at 850 MHz and 15.8 ms, 31.5 ms, 63.1 ms, 126.2 ms, 189.2 ms, and 252.3 ms at 500 MHz. Data were processed with nmrPipe,[9] apodized with a cosine squared bell function in the ^{1}H dimension and a cosine bell function in the ^{15}N dimension. Best-fit R_2 relaxation rates were calculated using least squares optimization of ^{1}H/^{15}N peak intensities to a single-exponential functions. ^{15}N R_2 was measured for 350 μM using matched samples (a 1 ml sample split into two 500 μl samples) simultaneously at 500 and 850 MHz ^{1}H frequencies, and ΔR_2 was calculated from the difference in ^{15}N-R_2 values at 350 μM and 50 μM concentrations.

$^{1}H_N$ transverse relaxation rates ($^{1}H_N$ R_2) were measured on samples of FUS LC at 350 μM and 50 μM at 500 MHz and 850 MHz ^{1}H frequencies with an interleaved experiment as described previously (Fawzi et al. 2010). Interleaved experiments comprised 128*×2048* complex data pairs in the indirect ^{15}N and direct ^{1}H dimensions, respectively, with corresponding acquisition times of 66 ms and 172 ms, sweep width of 19 ppm and 10.5 ppm, centered at 117 ppm and 4.9 ppm, respectively. Total R_2 relaxation delays were 0.2 ms, 10.2 ms, 20.2 ms, 40.2 ms, 60.2 ms, and 90.2 ms. As described above, data were processed with nmrPipe.

Particle tracking microrheology (PTM)

To perform PTM measurements of liquid condensates, Fluoresbrite®YG microspheres (1.00 µm) were pre-coated with PEG 400 in CAPS buffer, and then those beads were incubated in FUS LC at 4°C for 1h. Afterwards, condensates were created by mixing coated beads with protein with either a 4°C or room temperature phosphate buffer (~pH 7.4) to obtain a final concentration of 200 µM in pH 7.4. KTS condensates were obtained with a similar procedure as described above with a 4°C incubation time (overnight) while canonical FUS LC droplets were obtained by leaving the solution at room temperature for 10 minutes. Each sample was then drop cast on a coverslip and after 1 min of stabilization time, images were recorded with Mikrotron EoSens®QUAD1.1S camera; 250 fps in ROI of 256x256 pixels of GFP channel on an Olympus IX70 microscope. To exclude misleading data obtained for beads

attached to the edges of examined proteins condensates, the presence of beads inside droplets and KTS condensates was confirmed by manual Z scans in brightfield and fluorescence channels. Further processing of the video files was based on the use of Fiji software with the Track Mate plugin ver.6.0.1 that produced mean squared displacement (MSD) of bead tracks. The Trackmate algorithm was independently verified using a custom Python script based on TrackPy. Each movie was down-sampled to 50 fps to reduce computation time. MSD calculations were performed with bead tracks, which preserved their motion within the microscope focal depth of field for at least 90% of the track. Therefore, the displacement related to beads that went out of focus or flickered for more than 10% of the recording time was discarded for all samples and we could assume two-dimensional diffusion. At least 20 beads were tested for each sample – KTS condensates: 25 beads recorded in 6 movies, droplets: 23 beads recorded in 14 movies, buffer: 41 beads recorded in 3 movies. MSD was calculated for at least 18 s of time lag.

Molecular dynamics simulations

We used the Kim-Hummer (KH) coarse grained model,[10] which uses a one bead per amino acid level of resolution to represent the FUS LC 1-163 protein sequence in our CG simulations. As discussed in our previous work,[11] the potential energy of the system has contributions from electrostatic interactions between charged amino acids (Arg; Lys +1, Glu; Asp -1) represented by a Debye-Huckel functional form, bonded interactions between consecutive amino acids on the protein sequence represented by a harmonic potential and short-range interactions between amino acids were represented using a Ashbaugh Hatch functional form (Eqn S2-S5) with the interaction strengths informed by the statistical contact potential proposed by Miyazawa and Jernigan (MJ),[12]

$$\phi_{ij}(r) = \begin{cases} \phi_{ij}^{LJ}(r) + (1-\lambda_{ij})\epsilon, & r \le 2^{1/6}\sigma_{ij} \\ \lambda_{ij}\phi_{ij}^{LJ}(r), & r > 2^{1/6}\sigma_{ij}, \end{cases} \tag{S2}$$

where $\phi_{ij}^{LJ}(r)$ is the standard Lennard-Jones (LJ) potential shown below:

$$\phi_{ij}^{LJ}(r) = 4\epsilon\left[\left(\frac{\sigma_{ij}}{r}\right)^{12} - \left(\frac{\sigma_{ij}}{r}\right)^{6}\right]. \tag{S3}$$

and,

$$\epsilon = \left|\alpha(\epsilon_{MJ} - \epsilon_0)\right| \tag{S4}$$

$$\lambda = \begin{cases} 1 & \text{if } \epsilon_{MJ} \le \epsilon_0 \\ -1 & \text{otherwise} \end{cases} \tag{S5}$$

where ϵ_{MJ} is derived from the MJ statistical contact potential and ϵ_0 and α are parameters available in the KH model.

Using the HOOMD-Blue software package,[13] we simulated 100 chains of the FUS LC sequence in a 60 nm cubic box at a range of temperatures starting from a condensed condensate like configuration and radial density profiles were calculated for the protein and its constituent amino acids over a simulation time of ~5 microseconds as per protocol discussed in previous work.[11]

Supplemental Figures

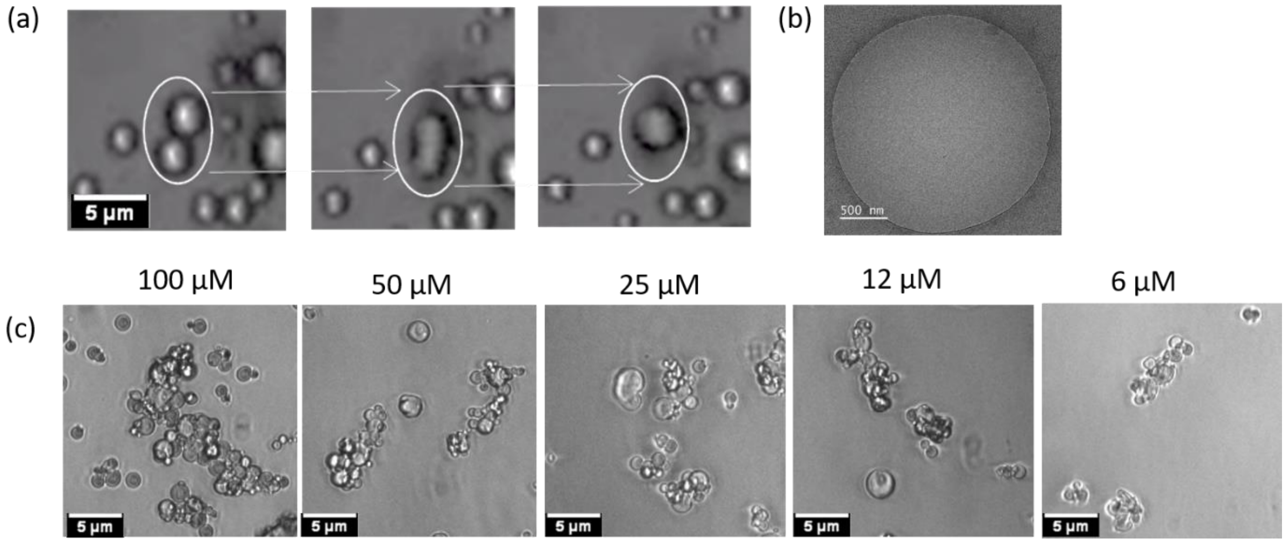

Fig S1. (a) bright field images of KUTS condensates exhibit dynamic liquid like properties, fusion and return to spherical morphology in pH 7.4 phosphate buffer containing 150 mM of NaCl. **(b)** Cryo-TEM imaging of KUTS condensates. **(c)** Bright field images KTS condensates under *in situ* (post formation) dilution from an original FUS LC concentration of 200 µM to the indicated FUS LC concentrations by adding pH 7.4 phosphate buffer containing 150 mM of NaCl.

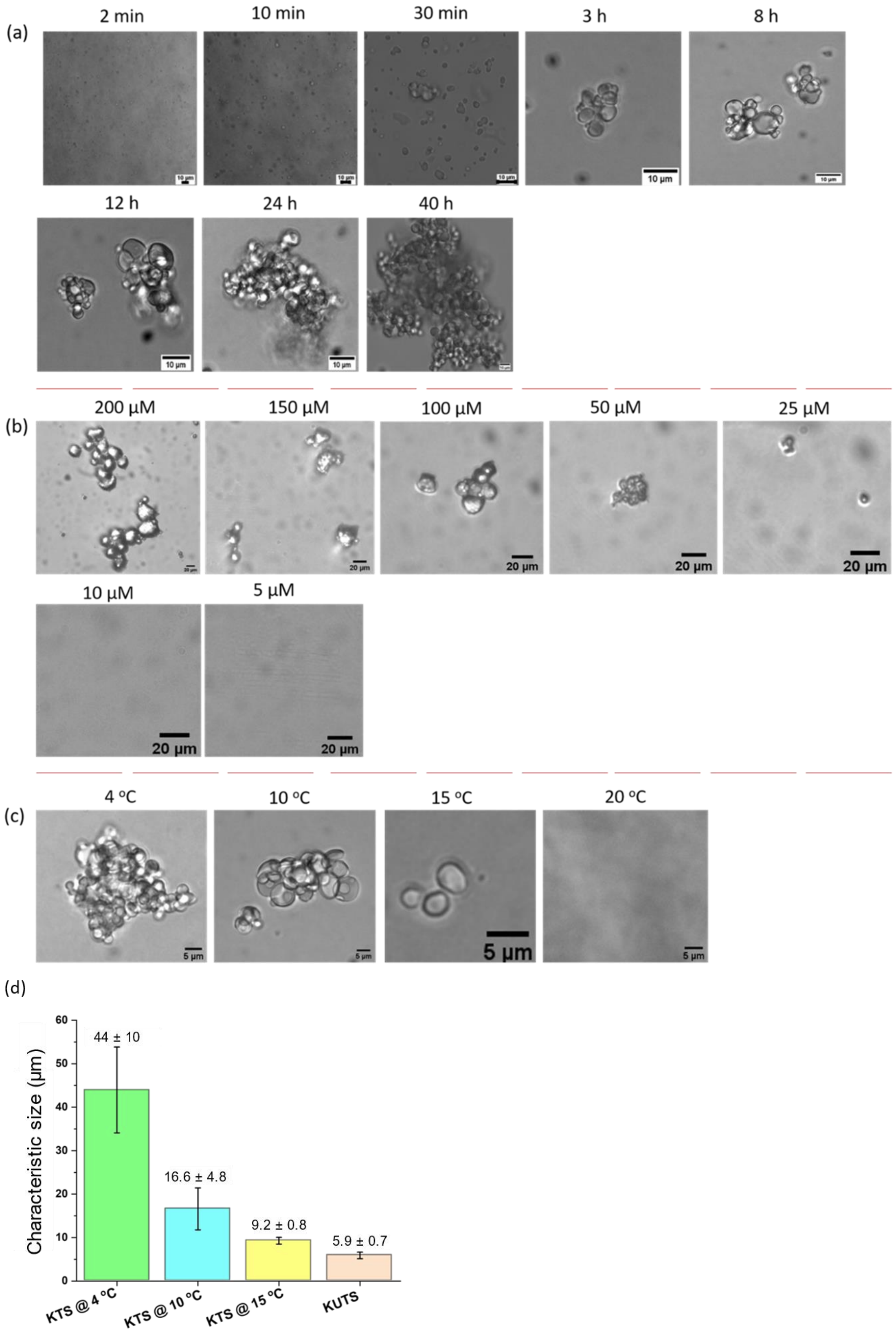

Fig S2. **(a)** cold formation (4 ^{o}C)-triggered KTS formation with different 4 ^{o}C incubation time as seen by brightfield microscopy. Images of the KTS at different time points indicated after cold formation the of the FUS LC segment (200 µM) in pH 7.4 phosphate buffer containing 150 mM of NaCl. **(b)** KTS formation by cold formation (4 ^{o}C) of different starting concentrations of FUS LC in pH 7.4 phosphate buffer containing 150 mM of NaCl. Brightfield images were taken at the indicated starting

concentrations and incubated for 12 hours at 4 ºC. **(c)** Formation of KTS by incubation of 200 µM of FUS LC at different temperatures for 12 hours (from left to right: 4 º to 20 ºC). Microscopy was performed at room temperature. **(d)** Size distribution showing the characteristic size of KTS condensates formed at the indicated temperatures overnight. The characteristic size distributions for KUTS condensate are shown for comparison. N ≥ 10 fields of view for all measurements; quantification is shown as mean ± standard deviation.

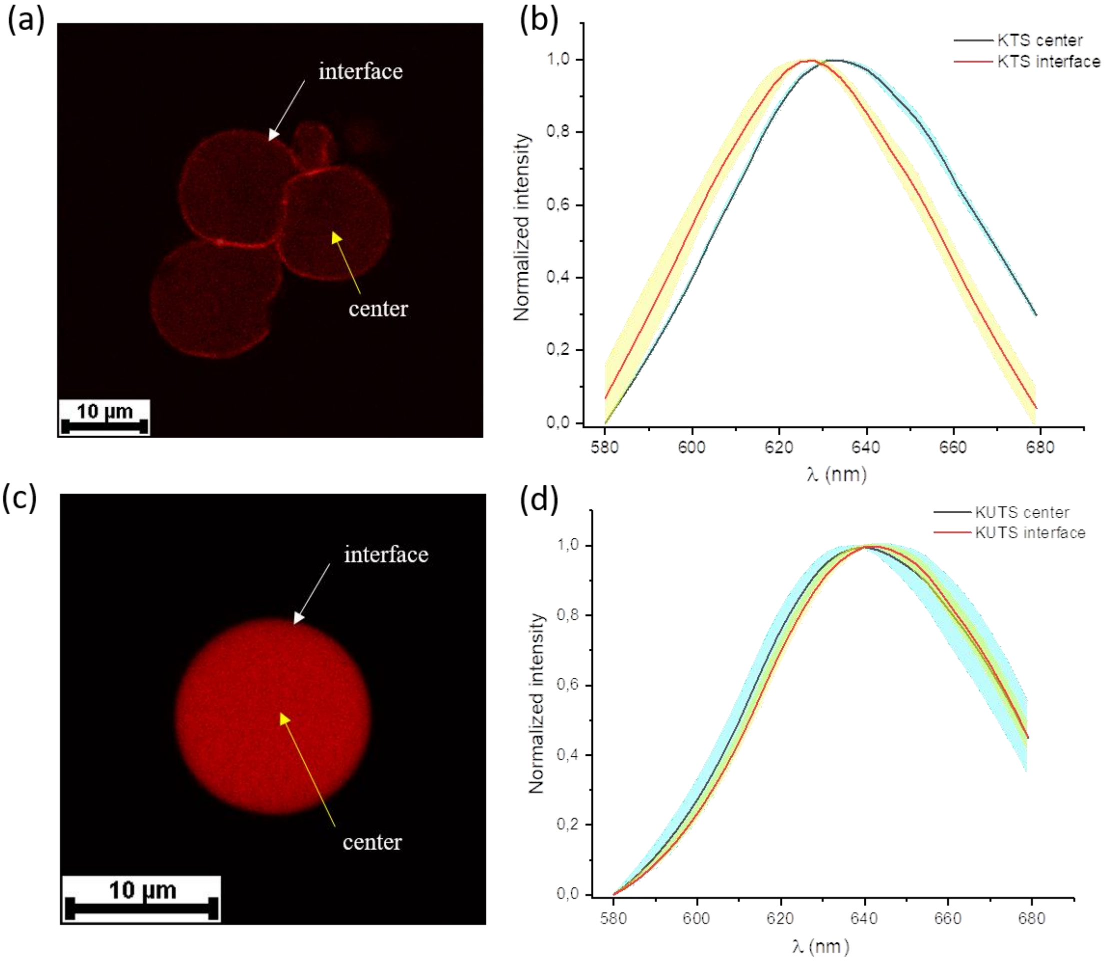

Fig S3. Confocal images of Nile red (20 µM) containing KTS **(a,b)** and KUTS **(c,d)** state using a 63X oil immersion objective (white and yellow arrows indicate the interface and the center of the KTS and KUTS samples). A room temperature stock solution of Nile red in DMF was added to the pre-formed KTS and KUTS condensates (with 200 µM FUS LC) in pH 7.4 phosphate buffer containing 150 mM of NaCl. Nile Red staining of the KTS and KUTS samples was done at room temperature, and spectral imaging was carried out at room temperature. A 514 nm laser was used to excite the Nile red dye and emission of this dye was monitored from 580 nm to 680 nm in the Leica SP5 system. This spectral data from the different scans was combined, average and normalized using Origin. The data showed different λ-emission maxima for the KTS sample at the interface and the center, while the shift in λ-emission maxima values at the center and at the interface of the KUTS sample are comparably much smaller.

Yellow and blue haze show the standard deviation from n = 7 spectral scans. Microscopy was performed at room temperature.

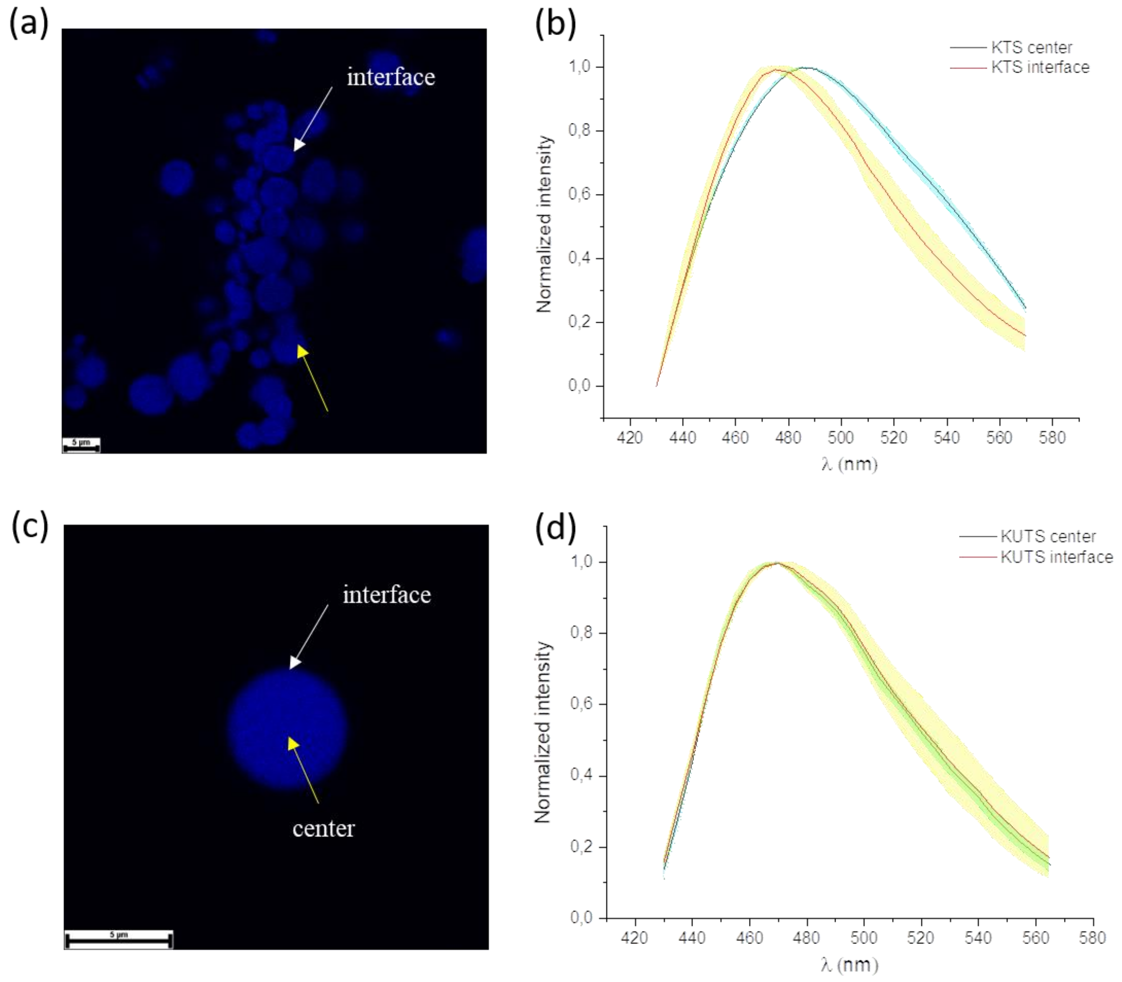

Fig S4. Confocal images of ANS (10 µM) containing KTS **(a,b)** and KUTS **(c,d)** state using 63X oil immersion objective (white and yellow arrows indicate the interface and the center of the KTS and KUTS sample). A room temperature stock solution of ANS in 20 mM of phosphate buffer (pH 7.4) was added to the pre-formed 200 µM of KTS and KUTS sample prepared in pH 7.5 phosphate buffer containing 150 mM of NaCl. ANS staining of the KTS and KUTS samples was done at room temperature, and spectral imaging was carried out at room temperature. A 405 nm laser was used to excite the Nile red dye and emission of this dye was monitored from 430 nm to 570 nm in the Leica SP5 system. Spectral data from the different scans was combined, average and normalized using Origin. The data showed different λ-emission maxima for the KTS sample at the interface and the center, while the similar λ-emission maxima values at the center and at the interface of the KUTS sample. Yellow and blue haze show the standard deviation from n = 7 spectral scans. Microscopy was performed at room temperature.

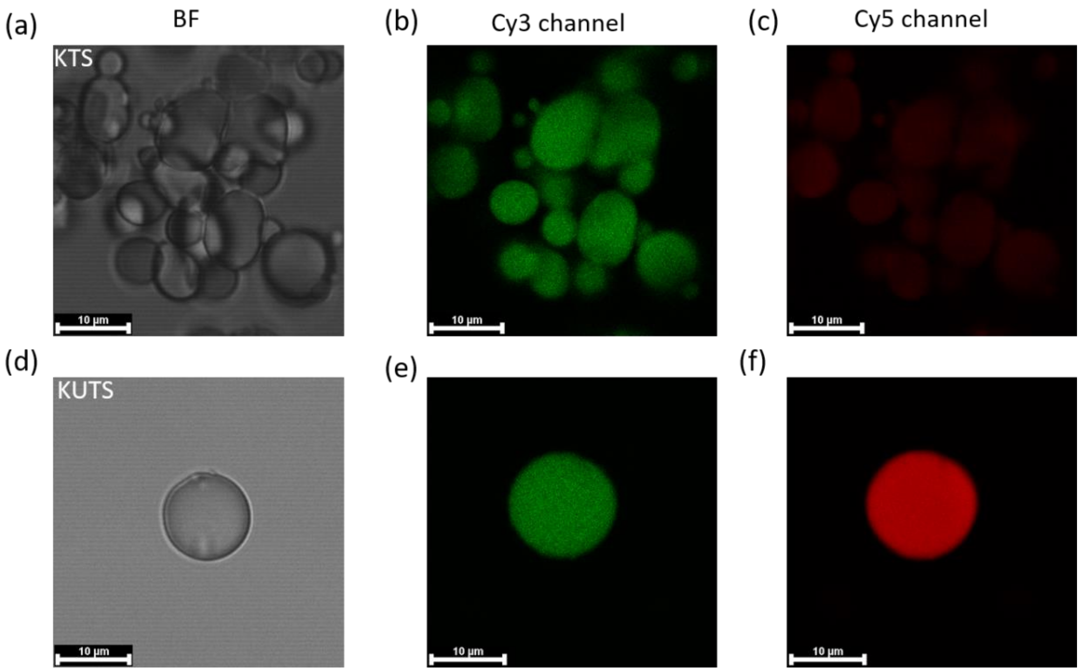

Fig S5. Bright field and fluorescence images (taken at room temperature) of Cy3-FUS LC (10 nM) doped into 200 µM unlabeled FUS LC during formation of KTS **(a, b)** and KUTS **(d, e)** condensates in pH 7.4 phosphate buffer containing 150 mM of NaCl. A 514 nm laser was used to excite the Cy3 dye. 5 nM of Cy5-FUS LC in CAPS (pH 11) was added *in situ* to the pre-formed Cy3-FUS and allowed to diffuse into KTS **(c)** and KUTS **(d)** condensates for 30 minutes. A 633 nm laser was used to excite Cy 5 dye. Identical acquisition and display settings were used for each respective channel across samples. Microscopy was performed at room temperature.

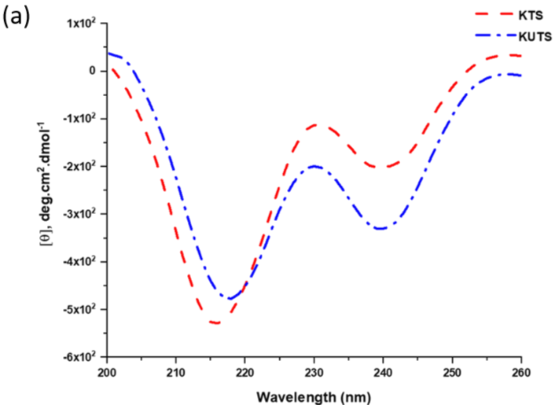

(b)

Sample	Helix (%)	Sheet (%)	Turn (%)	Other (%)
KTS	18.8	20.2	17.5	43.5
KUTS	23.3	16.1	16.9	43.7

Fig S6. (a) CD spectra of KTS (*in situ* diluted to 100 µM) and KUTS (*in situ* diluted 100 µM) in phosphate buffer containing 150 mM of NaCl (pH 7.4) at room temperature. CD spectra showed diverse spectral properties such as different amplitudes and wavelength in two different samples and **(b)** the

secondary structure composition of the two different states using spectral decomposition. Microscopy was performed at room temperature.

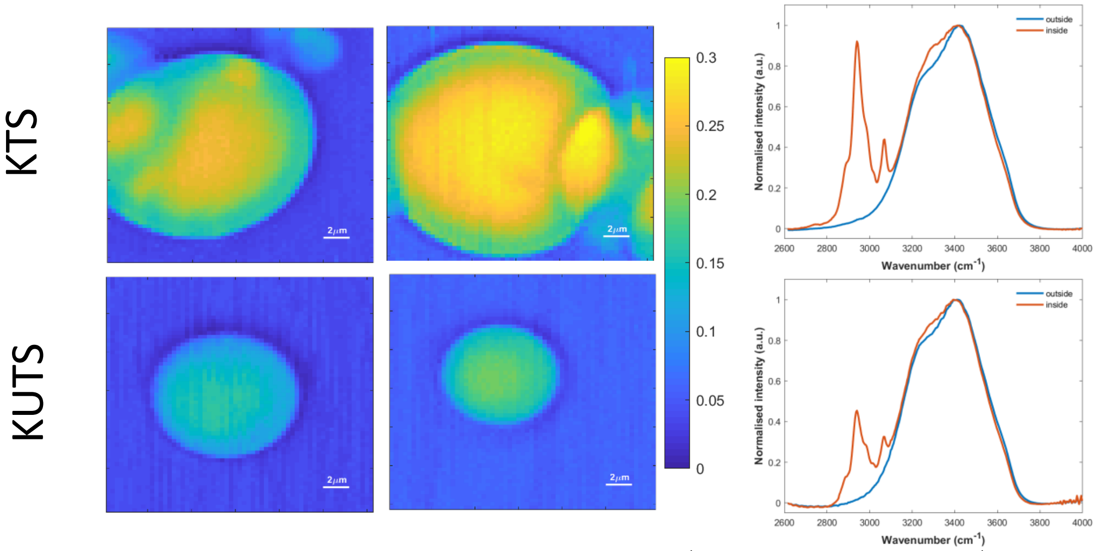

Fig S7. (*Left*) CARS ratio images of the CH (2800 -3100 cm^{-1}) to OH (3200-3700 cm^{-1}) range shows up to 2-fold more protein concentration in KTS condensates compared to KUTS condensates (two condensates for each type shown). The color bar and scaling are identical for all images. (Right) Averaged spectrum, normalized to OH intensity at 3400 cm^{-1}, with assigned buffer outside condensates showing mostly buffer (blue) and inside (red) KTS and KUTS condensates showing a strong CH peak.

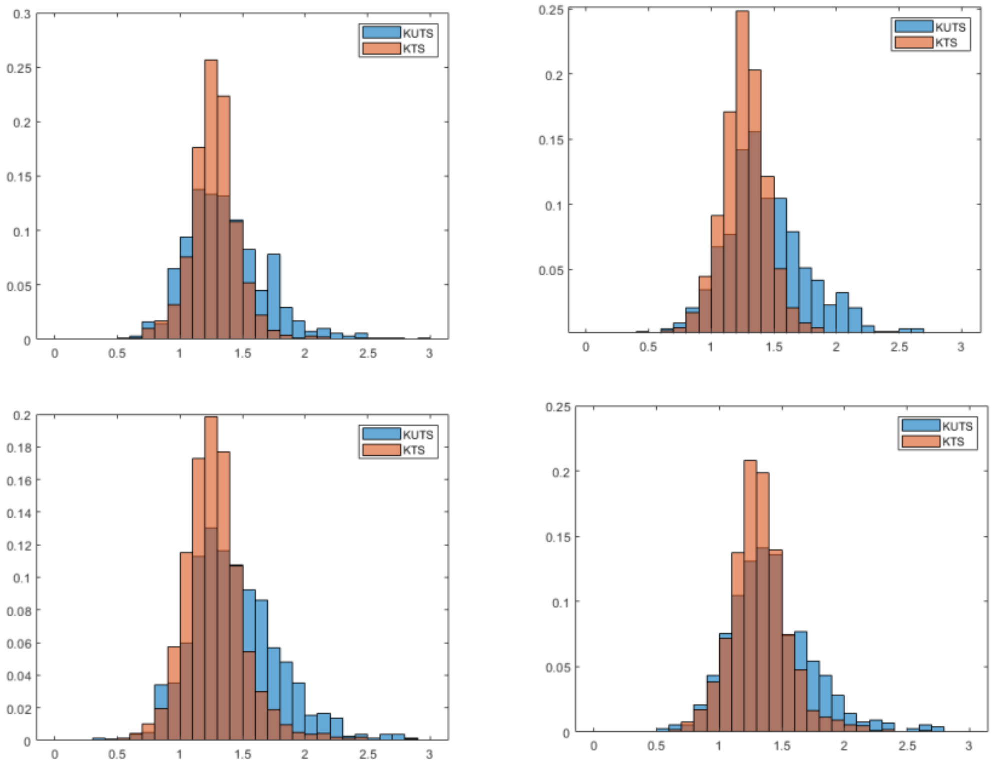

Fig S8. Tyrosine ratio histograms for pairwise comparisons between individual KUTS and KTS condensates. Values on the x-axis and frequency of occurrence on the y-axis.

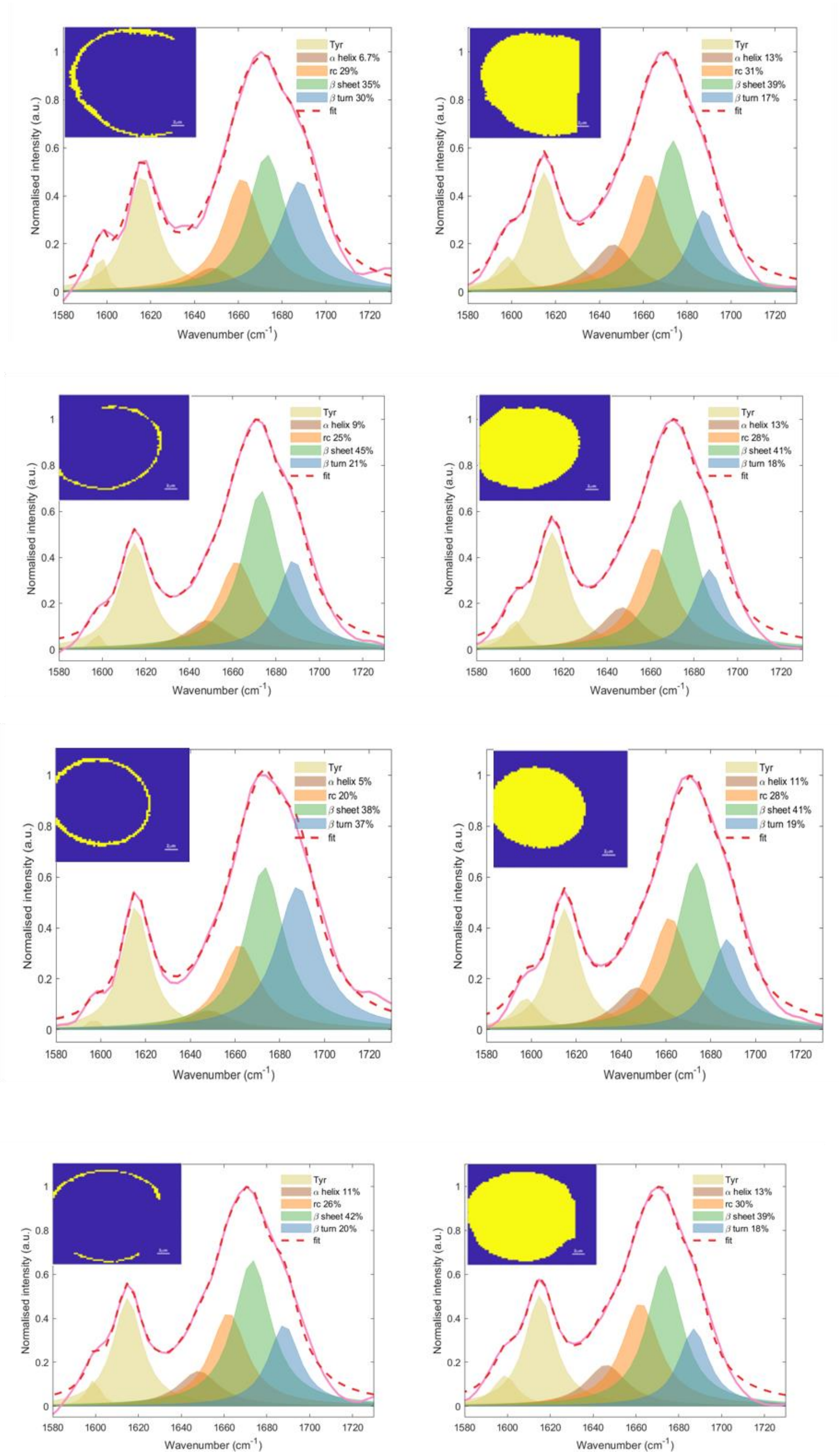

Fig S9. Amide I band fitting for independently prepared KTS (200 µM) sample in pH 7.4 phosphate buffer containing 150 mM of NaCl. ROI are shown in the insets. Scale bar 2 um. Microscopy was performed at room temperature.

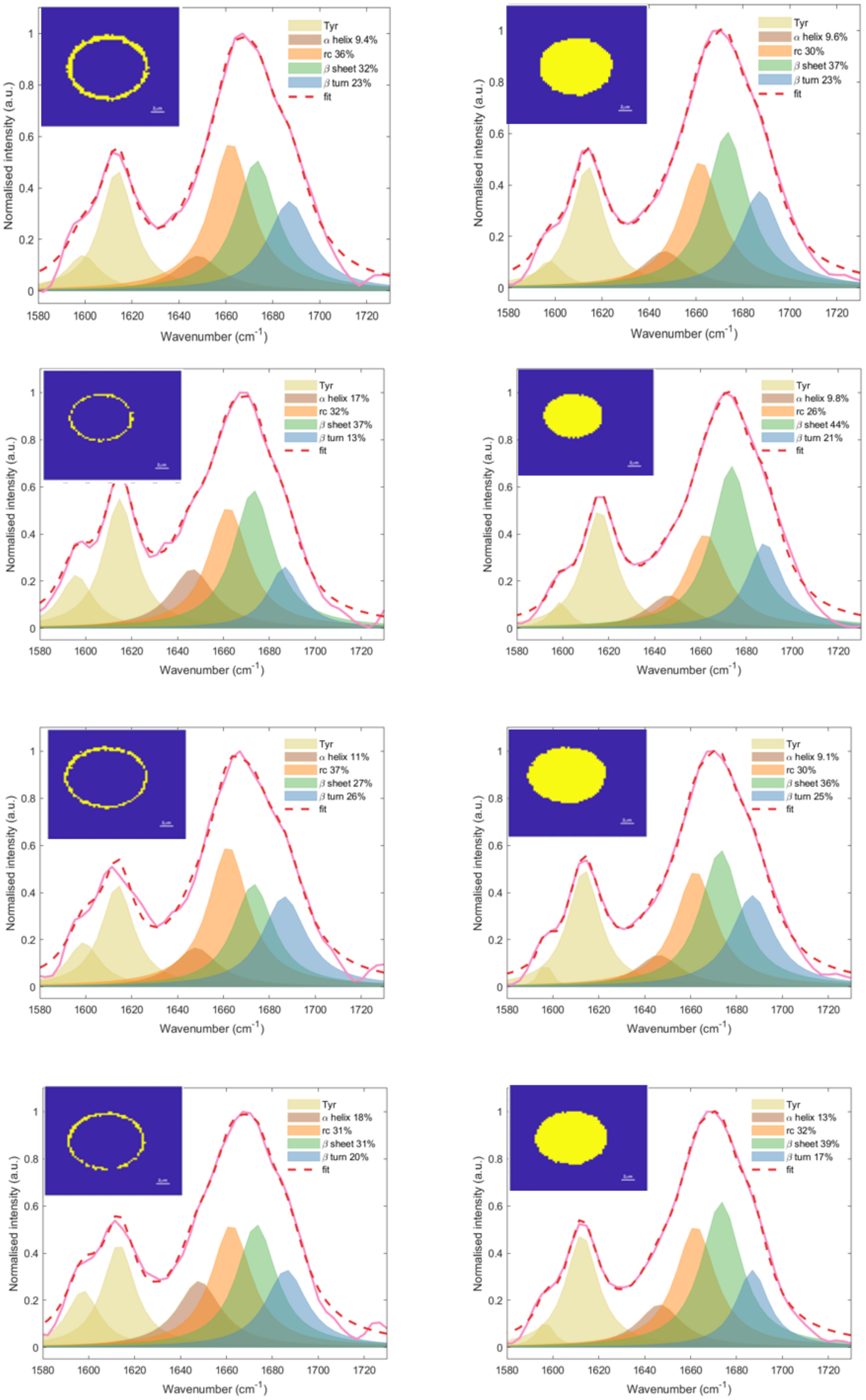

Fig S10. Amide I band fitting for independently prepared KUTS (200 μM) samples in pH 7.4 phosphate buffer containing 150 mM of NaCl. ROI are shown in the insets. Scale bar 2 um. Microscopy was performed at room temperature.

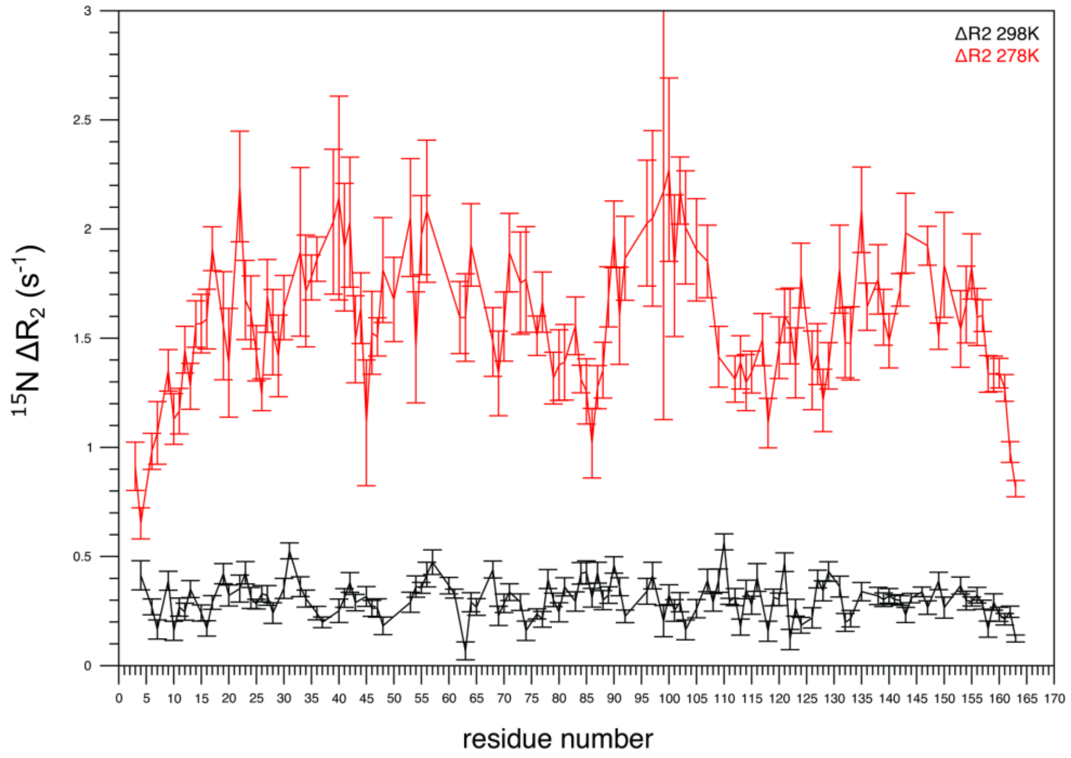

Fig S11. ^{15}N ΔR_2 (solid lines) measured by solution NMR are lower for room temperature droplets are performed at room temperature.lower always lower than 0.5 s^{-1} while those for cold droplets are generally above 1.5 s^{-1}.

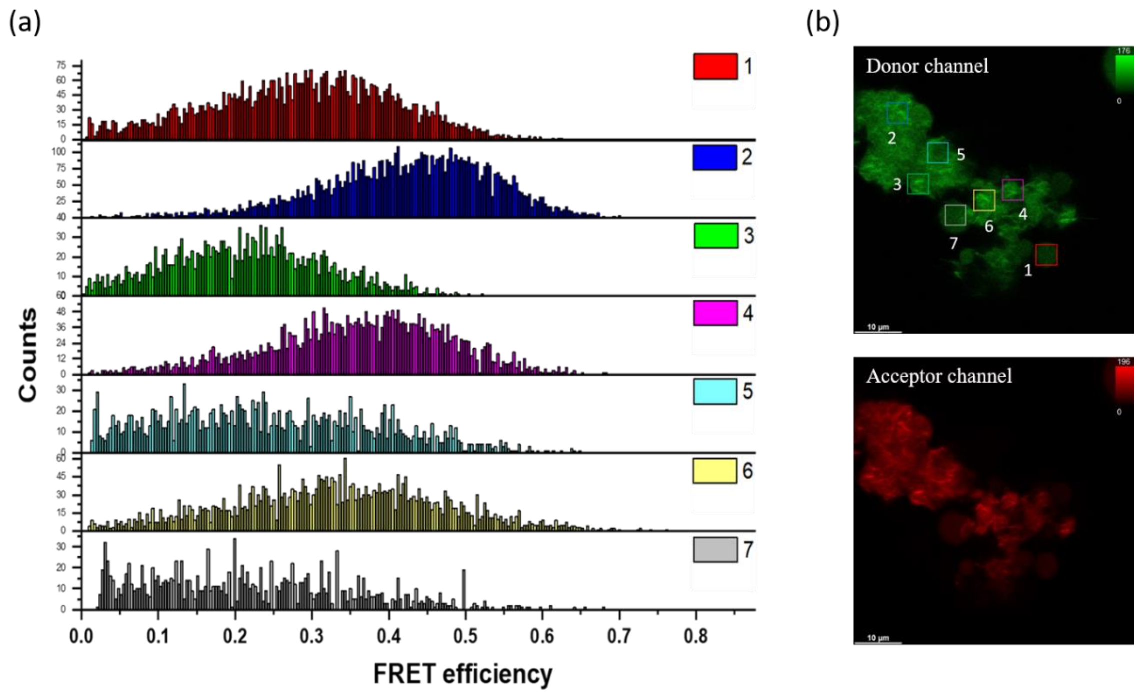

Fig S12. (a) Fluorescence resonance energy transfer efficiency (FRET *E*) histograms of the KTS (200 μM) samples by the acceptor bleaching (AB) method. Leica software was used to calculate the FRET efficiency at each point in a point-by-point manner according to $E =$

$(Donor\ Int_{post} - Donor\ Int_{pre})/Donor\ Int_{post}$, and **(b)** the regions of interest (ROIs) selected to calculate the FRET efficiencies shown in a for the donor and acceptor images. Microscopy was performed at room temperature.

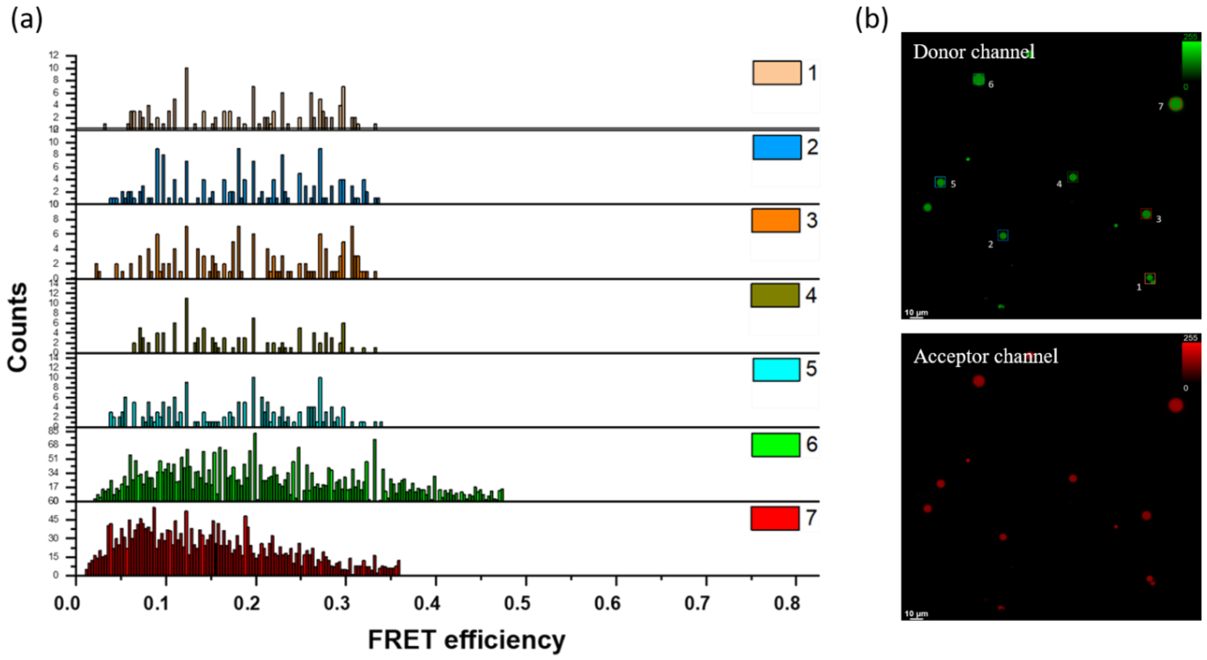

Fig S13. (a) FRET E histograms of the KUTS (200 μM) sample by the AB method. Leica software was used to calculate the FRET efficiency at each point in a point-by-point manner according to $E = (Donor\ Int_{post} - Donor\ Int_{pre})/Donor\ Int_{post}$, and **(b)** ROIs selected to calculate the FRET efficiencies shown in a for the donor and acceptor images. Microscopy was performed at room temperature.

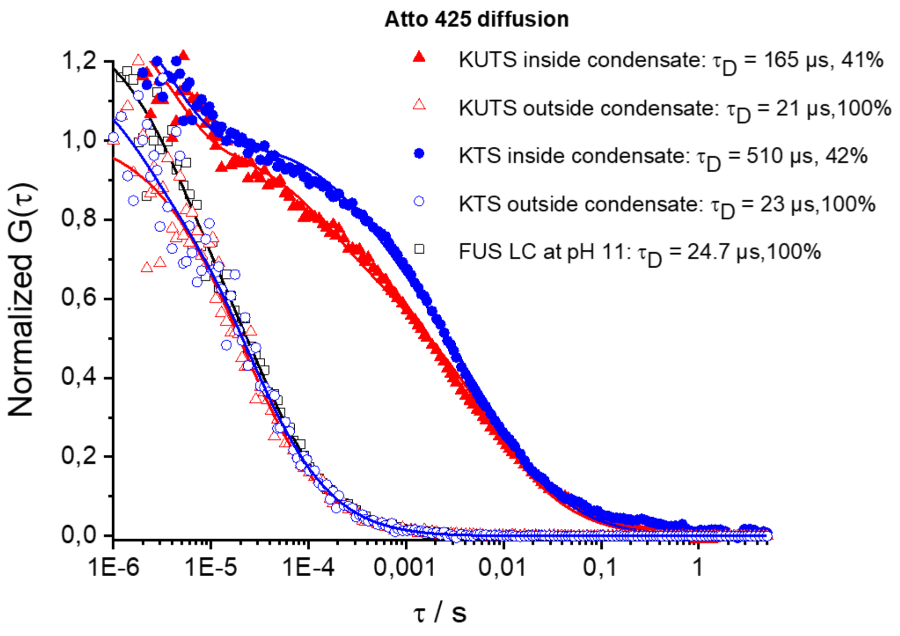

Fig S14. Normalized fluorescence correlation *G(τ)* curves of Atto 425 diffusion in KTS, KUTS and FUS LC at pH 11, respectively. All curves were fitted to Eq. 1 (inset, decay time values with contribution in different samples). Microscopy was performed at room temperature.

Table S1. Diffusion coefficient values of Atto 425 in different samples

Sample	Diffusion coefficient (m^2/s)
KTS inside condensate	1.57×10^{-11}
KTS outside condensate	34.3×10^{-11}
KUTS inside condensate	3.95×10^{-11}
KUTS outside condensate	37.6×10^{-11}
FUS LC at pH 11	31.9×10^{-11}

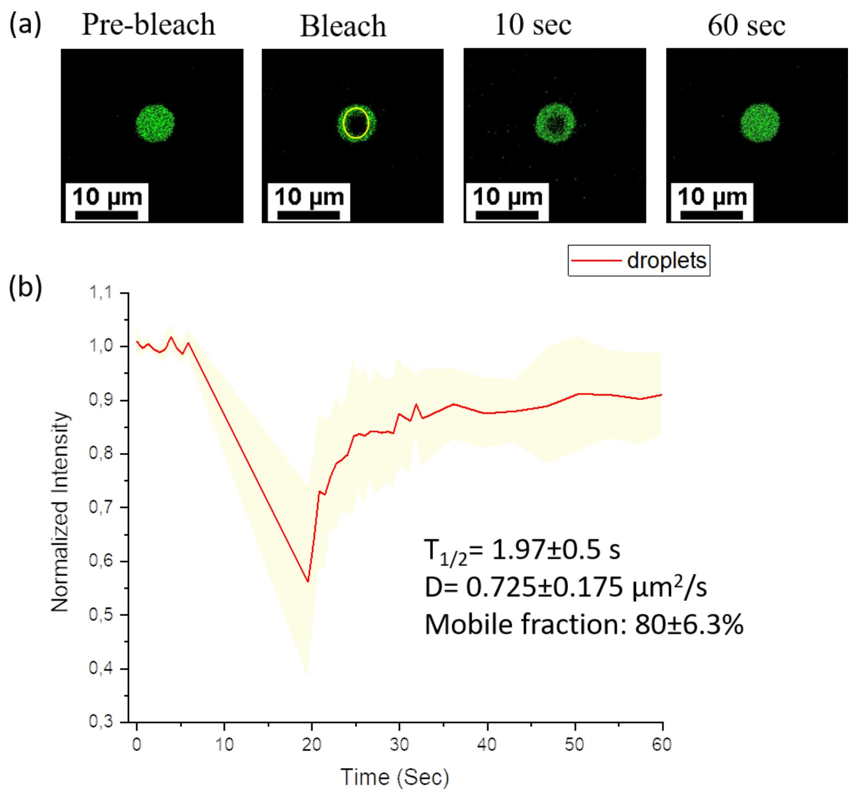

Fig S15. (a) Representative images of FRAP experiments with 0.01 mol % Cy3 labeled FUS LC in canonical FUS LC droplets by direct dilution of FUS LC into room temperature pH 7.4 phosphate buffer with 150 mM NaCl. The yellow circle highlights the punctum undergoing targeted bleaching. Images are labeled for their appropriate state with times being post bleaching. **(b)** Normalized fluorescence intensity over time, red line is the average over 6 samples with the standard deviation shown as yellow shading (inset, half-time fluorescence recovery ($T_{1/2}$), mobile fraction (%) distribution and diffusion coefficient values). Microscopy was performed at room temperature.

<u>Supplemental Movies</u>

Movie S1. Formation of KTS condensates from 5 to 16 minutes after buffer dilution using *in situ* microscopy with a temperature-controlled stage to ~ 4 ^{o}C.

References:

1 Billecke, N. *et al.* Perilipin 5 mediated lipid droplet remodelling revealed by coherent Raman imaging. *Integrative Biology* **7**, 467-476 (2015).

2 Liu, Y., Lee, Y. J. & Cicerone, M. T. Broadband CARS spectral phase retrieval using a time-domain Kramers–Kronig transform. *Optics letters* **34**, 1363-1365 (2009).

3 Koulouras, G. *et al.* EasyFRAP-web: a web-based tool for the analysis of fluorescence recovery after photobleaching data. *Nucleic acids research* **46**, W467-W472 (2018).

4 Burke, K. A., Janke, A. M., Rhine, C. L. & Fawzi, N. L. Residue-by-residue view of in vitro FUS granules that bind the C-terminal domain of RNA polymerase II. *Molecular cell* **60**, 231-241 (2015).

5 Wilkins, D. K. *et al.* Hydrodynamic radii of native and denatured proteins measured by pulse field gradient NMR techniques. *Biochemistry* **38**, 16424-16431 (1999).

6 Conicella, A. E. & Fawzi, N. L. The C-terminal threonine of Abeta43 nucleates toxic aggregation via structural and dynamical changes in monomers and protofibrils. *Biochemistry* **53**, 3095-3105, doi:10.1021/bi500131a (2014).

7 Fawzi, N. L., Ying, J., Ghirlando, R., Torchia, D. A. & Clore, G. M. Atomic-resolution dynamics on the surface of amyloid protofibrils probed by solution NMR. *Nature* **480**, 268-272, doi:10.1038/nature10577 (2011).

8 Fawzi, N. L., Ying, J., Torchia, D. A. & Clore, G. M. Probing exchange kinetics and atomic resolution dynamics in high-molecular-weight complexes using dark-state exchange saturation transfer NMR spectroscopy. *Nature protocols* **7**, 1523-1533, doi:10.1038/nprot.2012.077 (2012).

9 Delaglio, F. *et al.* NMRPipe: a multidimensional spectral processing system based on UNIX pipes. *Journal of biomolecular NMR* **6**, 277-293 (1995).

10 Kim, Y. C. & Hummer, G. Coarse-grained models for simulations of multiprotein complexes: application to ubiquitin binding. *Journal of molecular biology* **375**, 1416-1433 (2008).

11 Dignon, G. L., Zheng, W., Kim, Y. C., Best, R. B. & Mittal, J. Sequence determinants of protein phase behavior from a coarse-grained model. *PLoS computational biology* **14**, e1005941 (2018).

12 Miyazawa, S. & Jernigan, R. L. Residue–residue potentials with a favorable contact pair term and an unfavorable high packing density term, for simulation and threading. *Journal of molecular biology* **256**, 623-644 (1996).

13 Anderson, J. A., Glaser, J. & Glotzer, S. C. HOOMD-blue: A Python package for high-performance molecular dynamics and hard particle Monte Carlo simulations. *Computational Materials Science* **173**, 109363 (2020).
